# Trend of cancer risk of Chinese inhabitants to dioxins due to changes in dietary patterns: 1980–2009

**DOI:** 10.1038/srep21997

**Published:** 2016-02-25

**Authors:** Tao Huang, Wanyanhan Jiang, Zaili Ling, Yuan Zhao, Hong Gao, Jianmin Ma

**Affiliations:** 1 Key Laboratory for Environmental Pollution Prediction and Control, Gansu Province; College of Earth and Environmental Sciences, Lanzhou University, Lanzhou, 730000, China; 2 CAS Center for Excellence in Tibetan Plateau Earth Sciences, Chinese Academy of Sciences, Beijing, 100101, China

## Abstract

Food ingestion is a major route for human exposure and body burden to dioxins. We estimated the potential influence of changes in dietary patterns in Chinese population on human health risk to 2,3,7,8-TCDD (2,3,7,8-tetrachlorodibenzo-p-dioxin) over the last three decades. We performed multiple modeling scenario investigations to discriminate the contribution of 2,3,7,8-TCDD emissions and changes in dietary patterns to the cancer risks (CR) to dioxins. Results showed that changes in dietary patterns, featured by decreasing consumption of total grain (including all unprocessed grains) and vegetables and increasing intake of animal-derived foodstuffs, caused increasing CR from 7.3 × 10^−8^ in 1980 to 1.1 × 10^−7^ in 2009. Varying dietary patterns contributed 17% to the CR of Chinese population in 2009 under the fixed emission in 1980. The CR to 2,3,7,8-TCDD in urban and eastern China residents was higher considerably than those who lived in rural area and western China, attributable to higher emissions, household income, and greater intake of animal-derived foodstuffs in urban and eastern China inhabitants. On the other hand, more rapid increasing trend of the CR was found in rural residents due to their more rapid increase in the consumption of fat-dominated foods as compared with urban residents.

Polychlorinated dibenzo-p-dioxins (PCDD) and dibenzofurans (PCDF), commonly known as dioxins, are a lipophilic group of organic compounds, which are ubiquitous environmental pollutants^1^. Dioxins are produced unintentionally by human activities, such as waste incineration, chemical manufacturing, petroleum refining, fuel combustion in vehicles, wood burning, and electric power generation^2^. Dioxins have a toxicological profile similar to 2,3,7,8-tetrachlorodibenzo-p-dioxin (TCDD), the most toxic congener of dioxins. 2,3,7,8-TCDD (CAS Registry No: 1746-01-6, chemical formula C_12_H_4_Cl_4_O_2_) forms as stable by-product or contaminant during the production of 2,4,5-trichlorophenol (TCP) and in chlorophenoxy herbicides, including 2,4,5-trichlorophenoxyacetic acid (2,4,5-T) which were widely used in the 1960s and 1970s to control weeds and as a defoliant during the Vietnam War^3^. Dioxins can bind to (but not limited to) aryl hydrocarbon receptor (AhR) to exert a variety of toxicological effects, including dermal toxicity, developmental deficits, immunotoxicity, reproductive impairment, endocrine disruption, and carcinogenicity^4^. Once released into the environment, they can be transported globally and pose serious health and environmental risks. Due to their toxic properties and persistence in the environment^5^,^6^,^7^, the adverse effect of PCDD/Fs on human health and ecosystem has raised great concern in scientific and public communities.

Because of their high lipophilicity and low biodegradability, dioxins are also bioaccumulated in food chains, particularly in the fat-containing food^8^,^9^. Over 90% of human exposure to dioxins has been attributed to food consumption, followed by inhalation and dermal exposure to dioxins contaminated soils^10^. Fat-containing animal products and shellfish are major food sources of PCDD/Fs^11^. The dioxins traces have been detected in human tissue samples, such as breast milk, blood and adipose tissue^12^,^13^. China has been identified as the largest dioxin emitter in the world. Annual dioxin emission in China from 10 source groups was 10.2 kg TEQ in 2004, accounting for about 30% of global total emissions^14^,^15^.

Rapid economic development and increasing personal income over the last three decades has been leading to perhaps one of the most significant changes in food patterns in Chinese population in human history. Annual variation of dietary patterns and food item consumption from 1980 to 2009 in Chinese population can be found as Supplementary Fig. S1. As seen, per capita consumption of staple foods (namely unprocessed rice and wheat) has been declining during this period of time. The consumption of higher-value foods, especially the foods of animal origin, such as pork, beef, milk and dairy products, aquatic products, and poultry meats, on the other hand, is growing rapidly. Because these higher-value foods contain excessive amounts of fat, particularly saturated fat, and protein, animal-based diets have been linked to many of the chronic degenerative diseases that are characteristic of affluent societies, such as heart disease; colon, breast, and prostate cancer; and type II diabetes^16^. The increasing meat consumption in Chinese population is considered as a major cause for the increasing mortality induced by chronic diseases. It has been reported that measles, tuberculosis, and senility were the three most common causes of death before 1950. In 1985, malignant tumors, cerebrovascular disease, and ischemic heart disease were found to be the most common diseases responsible for the death of Chinese population^17^.

Due to technical difficulties in dioxin monitoring and laboratory analysis, information for dioxins contamination to food supply in China is extremely limited. This brings difficulties to assess health risk of dioxins in China. Models, with sufficient validation against monitored data, become useful for estimating the potential health risk caused by toxic chemicals, particularly in the assessment of long-term trend of health risks subject to changes in dietary patterns. Such the trend is almost unknown in China and worldwide.

Among PCDD and PCDF congeners, the levels and emissions of 2,3,7,8-TCDD in the environments across China are relatively lower as compared with some other congeners^18^. From the analysis of PCDD/PCDF profiles from large scale thermal processes and municipal solid waste incinerator (MSWI), Everaert and Baeyens have revealed the degree of chlorination points towards the dominant presence of HpCDD and OCDD within the dioxin group, and of PeCDF, HxCDF, and HpCDF within the furan group^19^. Zhang *et al.* examined 17 major dioxin congeners released from a large-scale MSWI in southern China and found that HpCDD and OCDD dominated total emission of dioxins^20^, agreeing with the result from Evraert and Baeyens^19^. However, given its highest toxicity and high lipophilicity, the present study will focus on assessments of the exposure risk of Chinese population to 2,3,7,8-TCDD. An atmospheric transport model and a food web model were combined to predict concentrations of 2,3,7,8-TCDD in various environmental media and major diets across China (Methods). Based on total exposure doses, the cancer risks (CR) of Chinese population due to exposure to 2,3,7,8-TCDD from 1980 to 2009 was evaluated using a health risk assessment model. The major objectives of this study were (1) to assess the effect and trend of the change in dietary patterns on human exposure risk to 2,3,7,8-TCDD in the past 30 years in China. (2) to fill data and knowledge gaps in human health and dioxin contamination to Chinese food web associated with rapid changes in the dietary patterns of Chinese population.

## Results and Discussion

### Linking change in dietary patterns with CR

To connect changes in dietary patterns with exposure risk of Chinese population to dioxins, extensive model simulations with 5 model scenarios were carried out using the combined atmospheric transport-food web-health risk assessment model (Methods). These 5 model scenarios are summarized in Supplementary Table S1. The first scenario incorporated the annual per capita food consumption and atmospheric 2,3,7,8-TCDD emission from 1980 to 2009 in China (scenario 1). The second scenario used a fixed emission inventory and fixed dietary patterns in 1980 (scenario 2). The third scenario considered the fixed emission inventory in 1980 and annually varying dietary patterns from 1980 to 2009 (scenario 3). The purpose of applying fixed emission inventory in the 2nd and 3rd model scenario was to tear off the influence of the change in dietary patterns on the human exposure risk to 2,3,7,8-TCDD from its annual emissions which would otherwise dominate annual variation of exposure risks. Given considerable differences in dietary patterns and personal income between urban and rural residents, the 4th and 5th model scenarios were set up to estimate the CR due to major food consumption in urban and rural residents, respectively (see Supplementary Table S1). Long-term trend of dietary patterns in urban and rural residents in China are illustrated in Supplementary Fig. S1(b,c). We also assessed the contribution from different dietary patterns in different regions to health risk. The dietary patterns of the residents in eastern, central, and western China were used in this health risk assessment. These three regions are specified in Supplementary Information (Section S2, Fig. S2), and the per capita food consumption in the three regions were shown in Supplementary Fig. S1(d–f).

### Temporal and spatial distribution of CR

**Figure 1:**
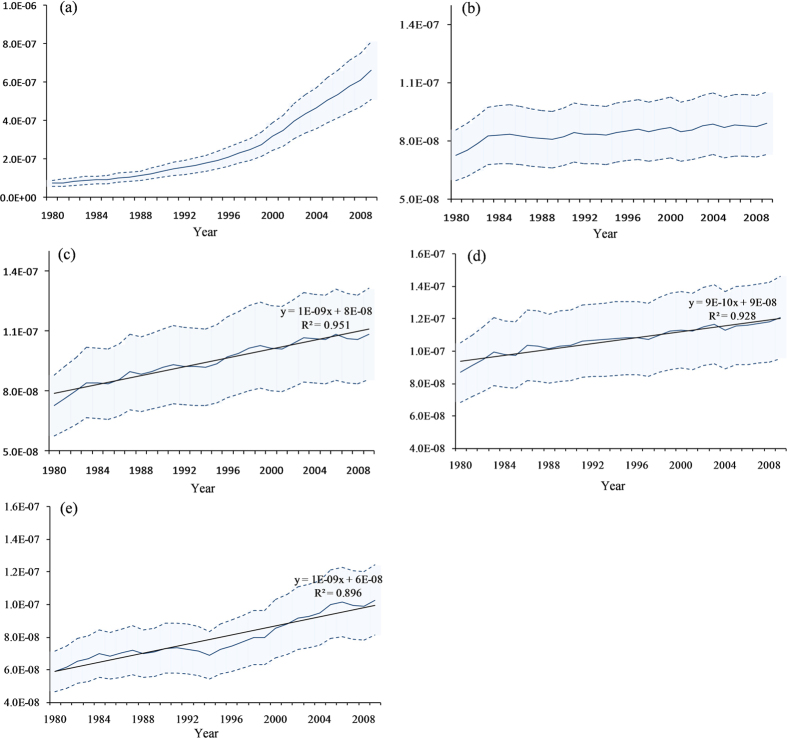
Spatially averaged CR over China due to human exposure to 2,3,7,8-TCDD through food intake in Chinese population from 1980 to 2009 (dimensionless), simulated from annual changes in emission of 2,3,7,8-TCDD and dietary patterns from 1980 to 2009 (scenario 1, **a**), fixed emission of 2,3,7,8-TCDD and dietary patterns in 1980 (scenario 2, **b**), fixed emission of 2,3,7,8-TCDD in 1980 and annual changes in dietary patterns in whole China from 1980 to 2009 (scenario 3, **c**), fixed emission of 2,3,7,8-TCDD in 1980 and annual changes in dietary patterns in urban residents from 1980 to 2009 (scenario 4, **d**), and fixed emission of 2,3,7,8-TCDD in 1980 and annual changes in dietary patterns in rural residents from 1980 to 2009 (scenario 5, **e**). Solid line represents the modeled CR using default input parameters and the shading (dashed lines) stands for 95% confidence interval.

Figure 1(a) shows the CR averaged over China due to human exposure to 2,3,7,8-TCDD through food consumption in Chinese population from 1980 to 2009, derived from the model scenario 1 (annual emissions of 2,3,7,8-TCDD and varying dietary patterns from 1980 to 2009). As shown, the averaged CR has increased almost one order of magnitude from 7.3 × 10^−8^ in 1980 to 6.6 × 10^−7^ in 2009 at an annually increasing rate of 28% (Table 1). In particular, the CR exhibited an dramatic increasing trend after 2000, attributable to more rapid increasing in 2,3,7,8-TCDD emissions since 2000 (see Supplementary Fig. S3). In China, iron and steel and other metal production industries were ranked as the biggest emission sources of dioxins, followed by waste incineration, and power and heat generation. Dioxins released from these three sources accounted for 83% of the total releases in China in 2004^14^. Due to rapid industrialization and urbanization of China over the past 30 years, the production of iron, crud steel, and electricity generation from coal power plants were reported to increase approximately 15, 16, and 12 fold as compared with that in 1980, respectively^21^,^22^. In the meantime, a large amount of municipal solid waste (MSW) have been also produced. As a result, the construction of municipal solid waste incinerators (MSWIs) has been booming since 2000^23^. These activities led to rapid incline of CR in China. Accordingly, population with higher CR exceeding the acceptable risk level of 10^−4^ (the U.S. EPA, Methods) also increased in the past 30 years (see Supplementary Fig. S4). By 2009, the Chinese population with CR to 2,3,7,8-TCDD exceeding the U.S. EPA acceptable risk level (1 × 10^4^) has reached 14.9 million (Table 1), accounted for approximately 10% of total Chinese residents.

Spatial distribution of the CR of Chinese population to 2,3,7,8-TCDD in 2009, derived from the model scenario 1 was shown in Supplementary Fig. S5(a). Higher CR can be found over eastern China due to the proximity of this part of China to major 2,3,7,8-TCDD emission sources. It has been reported that 2,3,7,8-TCDD emissions occurring in Beijing-Tianjin-Hebei region, Mid-north Liaoning, southeast seaboard, and East Sichuan Basin all together contribute approximately 68% to the national total emission in China in 2009^18^. CR values in some places over these regions exceeded the U.S. EPA acceptable risk level (1 × 10^4^). Low CR are identified in pristine regions with low population and far away from the emission sources.

To understand the overall CR in densely populated areas, population-weighted CR was calculated as the products of the predicted CR and population density divided by the national average population density. The calculated population-weighted CR is presented in Supplementary Fig. S5(b). High population-weighted CR values were mostly observed in Henan, the province with the highest population in China and those megalopolis in eastern China (e.g. Beijing and Tianjin), Shenyang in northeastern China, southeastern China (e.g., Guangzhou and Fuzhou), and cities in East Sichuan Basin due to spatial superimposition of the high CR level and high population density. Similar to population-weighted CR, population with higher cancer risk exceeding the acceptable risk level of 10^−4^ (the U.S. EPA) can also be found in the regions with higher 2,3,7,8-TCDD emission (see Supplementary Fig. S6).

### Effect of dietary patterns on CR

It should be noted that the trends and fluctuations of CR simulated from the first model scenario are dominated by annual 2,3,7,8-TCDD emissions (see Supplementary Fig. S3), rather than the changes in dietary patterns. To discern the contribution from the changes in dietary patterns of Chinese population to their health risk to dioxins, we performed two model scenarios simulation: 1. using fixed emission of 2,3,7,8-TCDD and dietary patterns in 1980 (model scenario 2) and 2. considering the fixed emission in 1980 and annually varying dietary patterns from 1980 to 2009 (model scenario 3). We then integrated model from 1980 to 2009. Results are displayed in Fig. 1(b,c). The spatially averaged CR from the model scenario 2 shows a rapid incline before 1984 but slowly increasing trend after 1984 subject to the fixed 2,3,7,8-TCDD emission and dietary patterns in 1980 (Fig. 1(b)). Since the model input initial concentrations of 2,3,7,8-TCDD in soil and water were set to zero, the modeled 2,3,7,8-TCDD levels in soil and water were increased quickly in the first several years of model integration until the chemical equilibrium between air and soil/water was reached. It is also noted that 2,3,7,8-TCDD emission in 1980 was implemented continuously in the model from 1980 to 2009, not merely in 1980. This resulted in slowly increasing trend of CR from 1980 to 2009, as shown in Fig. 1(b).

Figure 1(c) shows the spatially averaged CR over China simulated subject to model scenario 3. Statistically significant increasing trend is evident as compared with that derived from the model scenario 2 (Fig. 1(b)). Since in the model scenario 3, we took into consideration the fixed 2,3,7,8-TCDD emission in 1980 only, in this case, the emission would not make any contributions to the temporal trend of CR. The annual fluctuations and long-term trend therefore resulted mostly from changes in food ingestion. Result shows that food ingestion made the largest contribution to the CR (>95%) and was the major human exposure route to 2,3,7,8-TCDD, agreeing with previous studies^24^,^25^.

Benefited from remarkable economic growth since the early 1980s, substantial increasing personal income in Chinese population (see Supplementary Fig. S7(a)) resulted in significant changes in food supply, consumption and dietary patterns (see Supplementary Fig. S1(a)). The annual direct (unprocessed food) grain consumption per person dropped from 202 kg in 1980 to 135 kg in 2009, indicating a 33% reduction. The meat consumption (including pork, beef, mutton, poultry, and fish) has increased from 17 kg in 1980 to 37 kg in 2009 at an annual increasing rate of 4%. The increasing intake of animal-derived foodstuffs contaminated by 2,3,7,8-TCDD can also enhance the CR of Chinese population. Accordingly, the spatially averaged CR over China increased from 7.3 × 10^−8^ in 1980 to 1.1 × 10^−7^ in 2009, with annual increasing rate of 1.8% due to increasing intake of animal-derived foodstuffs (Table 1). Likewise, the population with CR exceeding the U.S. EPA acceptable level also increased from 1980 to 2009 subject to the model scenario 3 (see Supplementary Fig. S8). The contribution of the change in dietary patterns to CR was further assessed by calculating a cancer risk ratio *Con*_*diet*_ = (*CR*_*scenario 3*_/*CR*_*scenario 1*_) × 100 under the fixed (constant) air emission in 1980. Result indicates that the change in dietary patterns (mostly through intake of animal-derived foodstuffs) could approximately contribute 17% to the CR in 2009. However, when the rapid increasing of 2,3,7,8-TCDD emission during this period was taken into account, the contribution from the change in dietary patterns to CR dropped to 4% in 2009, implying a moderate influence of the varying dietary patterns on CR as compared with emission. This also suggests that, with deceasing dioxin emission in China in forthcoming years^26^,^27^, the dietary patterns in Chinese population will make an increasing contribution to their CR.

**Figure 2:**
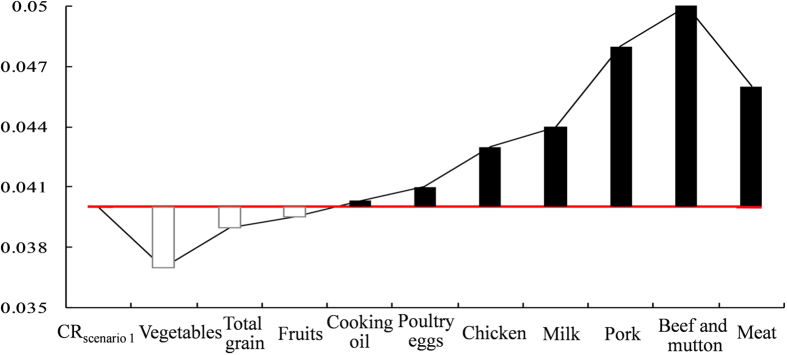
Critical CR frequency (=0.04, red solid line) derived from model scenario 1 and frequency of different food consumption from 1980 to 2009 deviated from the critical CR frequency, generated by spectral analysis.

Figure 2 shows the critical frequency of cancer risk (=0.04) derived from the model scenario 1, and the frequencies (occurrence) of CR from 1980 to 2009 derived from different food intake, calculated by a singular spectrum analysis method (see Methods). It can be seen that the frequencies of total grain, vegetables, and fruits were below the critical frequency of CR. On the other hand, the frequencies of meat, cooking oil, milk, and poultry eggs were well above the critical frequency of CR. This manifests that grains, vegetables, and fruits consumption decreased the probability of CR whereas the animal derived food consumption increased the probability of CR in China from 1980 to 2009. The beef and mutton contributed the maximum CR probability, followed by pork, milk, chicken, poultry eggs, and cooking oil, respectively.

**Figure 3:**
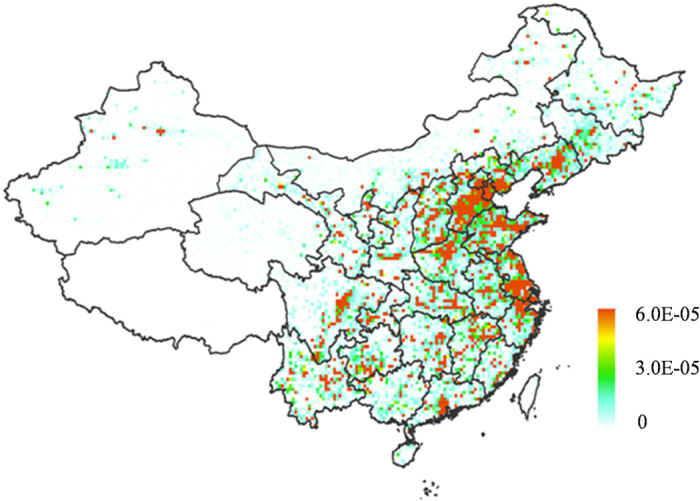
Difference of CR (ΔCR = CR_2009_–CR_1980_) between 1980 and 2009 subject to the model scenario 3 (fixed 2,3,7,8-TCDD emission in 1980 and annually varying dietary patterns from 1980 to 2009). The figure was generated by ArcGIS Desktop (version 10.2, ESRI, Redlands, USA).

Further insight into the effect of changes in dietary patterns on CR in Chinese population can be gained by comparing the CRs with their old and new dietary patterns. This was done by extracting the differences in the CRs (ΔCR = CR_2009_–CR_1980_) between 1980, when the dietary patterns was dominated by total grain, and 2009, when the animal-derived foodstuffs become a major part of the dietary patterns, notably in urban area (see Supplementary Fig. S1(b)). Figure 3 shows modeled gridded ΔCR across China. The model scenario 3 with the fixed 2,3,7,8-TCDD emission in 1980 and the annual dietary patterns from 1980 to 2009 was adopted in the model assessment. Higher ΔCR were found in Beijing-Tianjin-Hebei (BTH), Yangtze River Delta (YRD), Mid-north Liaoning, and East Sichuan Basin in eastern and central China due to the proximity of these regions to major emission sources in China^18^. This manifests that local residents in these regions would suffer from higher CR due to more significant changes in their dietary patterns than other regions across China.

There exist large imbalances in economic development and personal income between eastern China and other parts of the country. The per capita income in central and western China was lag behind eastern China considerably and such the income difference was expanding from 1980 to 2009 (see Supplementary Fig. S7(c)). Having higher purchase power of the residents from eastern China than central and western China residents, the consumption of higher-value food of animal origin, meats, poultry eggs, and milk in the residents of eastern China increased more rapidly as compared with that in central and western China. This also contributed more rapid increasing trend of CR in eastern China than central and western China (Fig. 3). With most dioxin-releasing industries in China^14^, higher population density^28^, and higher consumption of higher-value food of animal origin, the inhabitants in eastern China would suffer from higher CR to dioxin.

While the consumption of animal-derived foodstuffs per person in China has been growing rapidly over the last three decades, this consumption is still lower than many developed countries^29^,^30^. However, the exposure risk to 2,3,7,8-TCDD in Chinese population should not be overlooked, because the emission and environmental levels of this toxic chemical were much higher than developed countries. For instance, ambient PCDD/Fs air concentrations in many parts of China in the late 2000s were one-two orders of magnitude higher than their measured level in the United States^31^ and the United Kingdom^32^. This would lead to much higher dioxin contamination to the animal-derived foodstuffs in China, posing greater risk to Chinese population as compared with developed countries.

### CR by differences of dietary patterns between urban and rural residents

The household income has been considered as the most important factor affecting per capita food consumption^33^,^34^. Although the income of Chinese population as a whole has been growing rapidly over the last 3 decades, the income gaps between rural and urban residents in China have also been expanding during the same period of time. In 1980, the averaging income per capita in urban residents was 2.5 times higher than that of rural residents. In 2009, the income gap between urban and rural residents reached 2.9 times^21^,^22^.

As shown in Supplementary Fig. S1(b,c), urban residents with higher income consumed less total grain and vegetables but more animal-derived foodstuffs than the rural residents. In fact, the consumption of higher-value foods, especially foods of animal origin in urban residents increased from 31 kg in 1980 to 60 kg in 2009 whereas the rural residents consumed merely about 36 kg higher-value food in 2009, about 30 years behind the urban residents. The meat consumption (including pork, beef, mutton, poultry, and fish) per capita in urban residents enhanced from 26.3 kg in 1980 to 49.9 kg in 2009, rising 90% for this period of time. Whereas, per capita meat consumption in rural residents increased from 8.5 kg in 1980 to 24.9 kg in 2009, rising 193% for the same period. Both urban and rural residents doubled their consumption of cooking oils, eggs, and milk from 1980 to 2009. On the other hand, the direct grains consumption per person in urban residents dropped from 146 kg in 1980 to 81 kg in 2009, declining 45% over the three decades. During the same period of time, the unprocessed grains consumption in rural residents decreased by 26%, reducing from 257 to 189 kg^21^,^22^.

To assess the response of changing dietary patterns between urban and rural residents to CR, we estimated CRs of urban and rural residents induced by their exposure to 2,3,7,8-TCDD subject to changes in their respective dietary patterns. The model simulations were carried out subject to the model scenarios 4 and 5 (fixed emission in 1980 and annually varying dietary patterns from 1980 to 2009) for urban and rural residents, respectively. Results are displayed in Fig. 1(d,e). Due to increasing consumption of fat-containing animal products, CR in both urban and rural residents increased during the period of 1980 through 2009. Given large difference in food consumption patterns, the urban and rural residents also exhibited significant difference in exposure risk to 2,3,7,8-TCDD. As shown in Supplementary Fig. S9(d,e), the percentage of the risk via ingesting fat-containing animal products such as meat, eggs and milk in urban residents is much higher than rural residents. This difference leads to higher CR in urban residents, as shown by Fig. 1(d). However, due to more rapid increase in the intake of animal products in rural residents, our modeling result also indicated that the CR in rural residents from meat consumption along increased from 18% in 1980 to 40% in 2009, with 22% incline over the 30 years period, as compared with 13% increase in urban residents for the same period of time. As a result, while the similar spatial pattern of the CR difference between 1980 and 2009 in urban and rural residents was observed, this difference in rural residents has a greater slope (1 × 10^−9^) than that in urban residents (1 × 10^−10^) due to faster increase in their meat intake (Fig. 1(d,e)).

**Figure 4:**
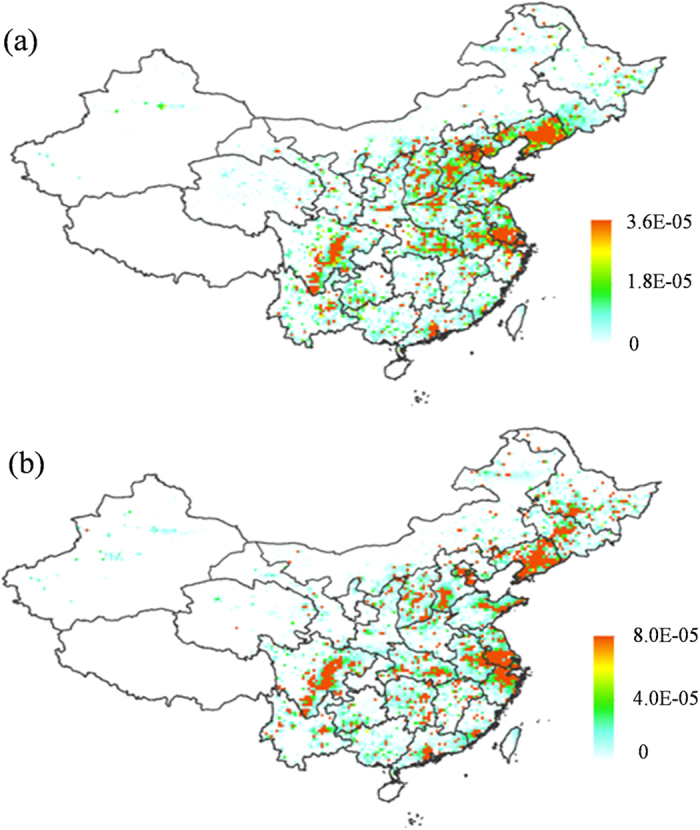
Differences of CR induced by exposing to 2,3,7,8-TCDDD between 1980 and 2009 from (**a**) scenario 4 (constant 2,3,7,8-TCDD emission in 1980 and annually varying dietary patterns in urban area from 1980 to 2009); (**b**) scenario 5 (constant 2,3,7,8-TCDD emission in 1980 and annual dietary patterns in rural area from 1980 to 2009). The figure was generated by ArcGIS Desktop (version 10.2, ESRI, Redlands, USA).

As expected, the more rapid increasing in meat consumption in rural residents yields greater CR difference between 1980 and 2009, as shown in Fig. 4. However, this does not suggest that the rural residents would suffer from higher CR than the urban residents. As shown in Fig. 1(d,e), overall the CR values in the urban residents were greater than the rural residents. By 2010, the urban population reached almost 50% of the national total population. It is expected that the urbanization would request increasing demand and supply of animal products and other higher valued foods. As a result, the urbanization in China is likely to enhance potentially the exposure risk of Chinese population to dioxins if the emission of this toxic chemical in China dose not decline significantly in forthcoming years.

## Conclusion

This study assessed the effect of the changes in dietary patterns in Chinese population on their exposure risk to 2,3,7,8-TCDD for the past three decades. Due to rapid increasing in consumption of animal-derived foodstuffs, the annually averaged CR increased from 7.3 × 10^−8^ in 1980 to 1.1 × 10^−7^ in 2009 under the condition of fixed emission in 1980 and annually varying dietary pattern from 1980 to 2009. We showed that 2,3,7,8-TCDD atmospheric emissions made major contribution to the CR levels and dominated long-term trend in China. In the case of fixed emissions, however, annually varying dietary patterns could account for 8% of CR of Chinese population in 1980 and 17% in 2009.

We found that the CR to 2,3,7,8-TCDD were higher in urban area and eastern China than rural and western China. This is attributed to stronger dioxin emissions, greater consumption of animal-derived foodstuffs, and higher population density in urban area and eastern China. With rapid urbanization and immigrations of Chinese people to developed regions in eastern China, increasing population in urban areas and developed regions might lead to increasing consumption of animal products, which, in turn, might enhance further exposure risk to dioxins of Chinese population under current emission levels in China. It is expected that, with decreasing dioxin emissions in China in forthcoming years, the current dietary patterns in Chinese population will make an increasing contribution to their CR to dioxins.

## Methods

The model framework applied in the present study is a combination of three models including an atmospheric transport model, a food web model, and a health risk assessment model. These three models are briefly elaborated below.

### Atmospheric Transport Model

2,3,7,8-TCDD concentration levels in air, soil, and water as well as its atmospheric transport and deposition were predicted by a modified version of the CanMETOP (Canadian Model for Environmental Transport of Organochlorine Pesticides)^35^,^36^ and using a high spatial resolution emission inventory of 2,3,7,8-TCDD from 1980 to 2009^18^ (see Supplementary Information Section S4). The primary features of the model are the same as the global version of CanMETOP^34^ but the horizontal spatial resolution was reduced to 1/4° latitude by 1/4° longitude with 14 vertical layers from 0, 1.5, 3.9, 10, 100, 350, 700, 1200, 2000, 3000, 5000, to 7000, 9000, and 11,000 m. Three dimensional atmospheric advection, eddy diffusion, dry/wet depositions, gas-particle partitioning, and the removal processes in air, soil, and water via degradation processes were implemented in the model. A dynamic, three soil layers, fugacity-based mass balance model and a two-film model were coupled with the CanMETOP to estimate diffusive soil-air and water-air gas exchange. The model domain covers China and surrounding regions (see Supplementary Fig. S10). The model was integrated from 1980 to 2009 to simulate long-term trend of 2,3,7,8-TCDD in multiple environmental compartments subject to different model scenarios. Detailed model descriptions can be found elsewhere^35^,^36^,^37^,^38^, and the model parameterization and validation were provided in Supplementary Information (Section S8, Figs S3, S11–S13, Tables S2–S5).

### Food Web Model

Using CanMETOP simulated 2,3,7,8-TCDD concentrations in air, airborne particles, soil, and water as the food web model inputs, 2,3,7,8-TCDD levels in foods were estimated using a food web model. In this model, 2,3,7,8-TCDD concentrations in fish was calculated using a level III fugacity module^39^. 2,3,7,8-TCDD accumulation in plants was estimated by integrating the processes of dry gaseous atmospheric deposition, wet and dry particle atmospheric deposition, and root uptake from soil and translocation within the plants^40^. Beef, pork, and chicken were prevailing raw meats for Chinese residents and considered as meat in the model. 2,3,7,8-TCDD levels in livestock, milk, and egg were cascaded up from its concentration in animal’s diet by using a bioconcentration model^40^. Detailed descriptions on food web model and evaluation were presented in Supplementary Information (Section S9, Figs S14 and S15, Tables S6–S9).

### Health risk assessment model

Ingestion, inhalation, and dermal contact with contaminated substances are main routes for human exposure and body burden to dioxins^41^. 14 possible exposure pathways, including inhalation through air and airborne particles, ingestion of cereal, vegetable, edible oil, fruit, fish, meat, milk, egg, and drinking water, as well as dermal contact with airborne particles, soil, and water were taken into consideration in this modeling assessment. We hypothesized that the human exposure risk to dioxins at each model grid point was induced primarily by 2,3,7,8-TCDD contamination to the local environments and foods. Although foods might be transported from one place to another, Chinese residents consumed mostly the locally growing foods^42^. The total exposure doses (TEDs, pg/kg bw/day) were estimated by the sum of the intakes via inhalation, ingestion, and dermal exposure. The CR caused by 2,3,7,8-TCDD was estimated from TEDs multiplied by a cancer slope factor. For toxicity pathways, the intake via inhalation and dermal contact were assumed to be equivalent to the oral intake. A cancer slope factor of 1.0 × 10^−3^ (per mg kg^−1^ day^−1^) for oral intake of 2,3,7,8-TCDD, proposed by the U.S. EPA^43^, was used to every exposure pathway. The detailed information on CR is presented in Supplementary Information (Section S10, Table S10). It should be noted that, although risk assessment methods for carcinogens have been improved over the past two decades^44^,^45^, quantitative health risk assessments for carcinogenic contaminants still exist uncertainties and possibly intractable methodological problems and challenges^46^. One of such uncertainties is the determination of an acceptable risk level for human exposure to a toxic contaminant^47^,^48^,^49^. In the present study, the acceptable CR of 10^−4^ recommended by the U.S. EPA was adopted^49^.

### Spectral Analysis

Spectral (frequency domain) method has been widely used to discern the signals in a time series which may contain important frequency information of the time series. Valachovic *et al.* have used the spectral method to examine the long term changes in skin cancer and sunspot number as a proxy for total solar irradiance and the relationship between solar cycle and skin cancer^50^. The detailed descriptions of spectral analysis can be found elsewhere^51^,^52^. In the present study, to examine the influence of dietary patterns on human exposure risk to 2,3,7,8-TCDD, the (occurrence) frequencies of cancer risk derived from scenario 1, and various food consumption from 1980 to 2009 was calculated by a singular spectrum analysis tool developed by Jannis^53^.

### Uncertainty evaluation

Monte Carlo analysis was conducted to quantify the uncertainties in health and environmental risk assessments^54^. Monte Carlo analysis requires parameterization of the degree of uncertainty and the shape of input distributions that are appropriate to each individual input parameter. Due to broad regional scale and a large number of input parameters, detailed parameterization of the distributions may be difficult. In the present study, the first-order error propagation derived by Macleod *et al.* was used to calculate the uncertainties in modeled concentrations (*Cf*_*out*_) generated from the uncertainties in input parameters (*Cf*_*i*_), given by^55^


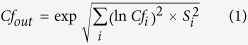


where *Cf*_*out*_ and *Cf*_*i*_ are the confidence factors that span the 95% confidence interval around the median of a log-normally distributed variable, and *S*_*i*_ is the relative sensitivity of the model output toward a change in input parameter *i*.

The *Cf* of input parameter uncertainties can be calculated as follows:^55^


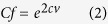


where *CV* is coefficient of variation, defined as the ratio of the standard deviation to the mean.

The sensitivity (*S*) is calculated by


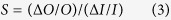


where Δ*I* and Δ*O* are the relative changes in input (*I*) and output (*O*) parameters of interest, respectively. The average sensitivity of increasing and decreasing input parameter was calculated. Each input parameter was varied individually by ±10%, respectively.

For unavailable CV, the input parameter (e.g., dioxins properties) uncertainties were assigned *Cf*_*s*_ default values recommended by Macleod *et al.*^55^ and Wöhrnschimmel *et al.*^56^. The uncertainties of model input parameters used in three models were presented in Supplementary Information (Tables S2, S7 and S10).

## Additional Information

**How to cite this article**: Huang, T. *et al.* Trend of cancer risk of Chinese inhabitants to dioxins due to changes in dietary patterns: 1980–2009. *Sci. Rep.* **6**, 21997; doi: 10.1038/srep21997 (2016).

## Supplementary Material

Supplementary Information

## Figures and Tables

**Table 1:** Human risk of Chinese residents induced by exposing to 2,3,7,8-TCDD derived from different modeling scenarios.

		scenario 1	scenario 2	scenario 3	scenario 4	scenario 5
Cancer risk (dimensionless)	1980	7.3 × 10^−8^	7.3 × 10^−8^	7.3 × 10^−8^	8.7 × 10^−8^	5.9 × 10^−8^
	2009	6.6 × 10^−7^	8.9 × 10^−8^	1.1 × 10^−7^	1.2 × 10^−7^	1.0 × 10^−7^
	Annual average increasing rate (%)	27.7	0.76	1.75	1.31	2.98
Population with higher cancer risk (⩾10^4^) (million)	1980	1.4	1.4	1.4	1.8	1.1
	2009	14.9	1.7	2.2	2.5	2.1
	Annual average increasing rate (%)	33.2	0.74	1.97	1.34	3.13
Confidence factor (CF) of cancer risk		1.6	1.3	1.4	1.4	1.4
