# Supplementary material for: Trend of cancer risk of Chinese inhabitants to dioxins due to changes in dietary patterns: 1980–2009
Source: Sci Rep. 2016 Feb 25;6:21997. doi: 10.1038/srep21997 (PMC4766489; doi:10.1038/srep21997)
Supplement: Supplementary Information [file srep21997-s1.pdf]

Supplementary Information for

Trend of cancer risk of Chinese inhabitants to dioxins due to changes in dietary patterns: 1980-2009

Tao Huang[1*], Wanyanhan Jiang[1], Zaili Ling[1], Yuan Zhao[1], Hong Gao[1], Jianmin Ma[1,2*]

[1]Key Laboratory for Environmental Pollution Prediction and Control, Gansu Province; College of Earth and Environmental Sciences, Lanzhou University, Lanzhou, 730000, China

[2]CAS Center for Excellence in Tibetan Plateau Earth Sciences, Chinese Academy of Sciences, Beijing 100101, China

Corresponding author: Jianmin Ma, Tao Huang

College of Earth and Environmental Sciences, Lanzhou University, 222, Tianshui South Road, Lanzhou 730000, China

Tel: (86) 152-9316-6921

Fax: (86) 931-891-1843

Email: jianminma@lzu.edu.cn, huangt@lzu.edu.cn

S1 Food consumption of Chinese population

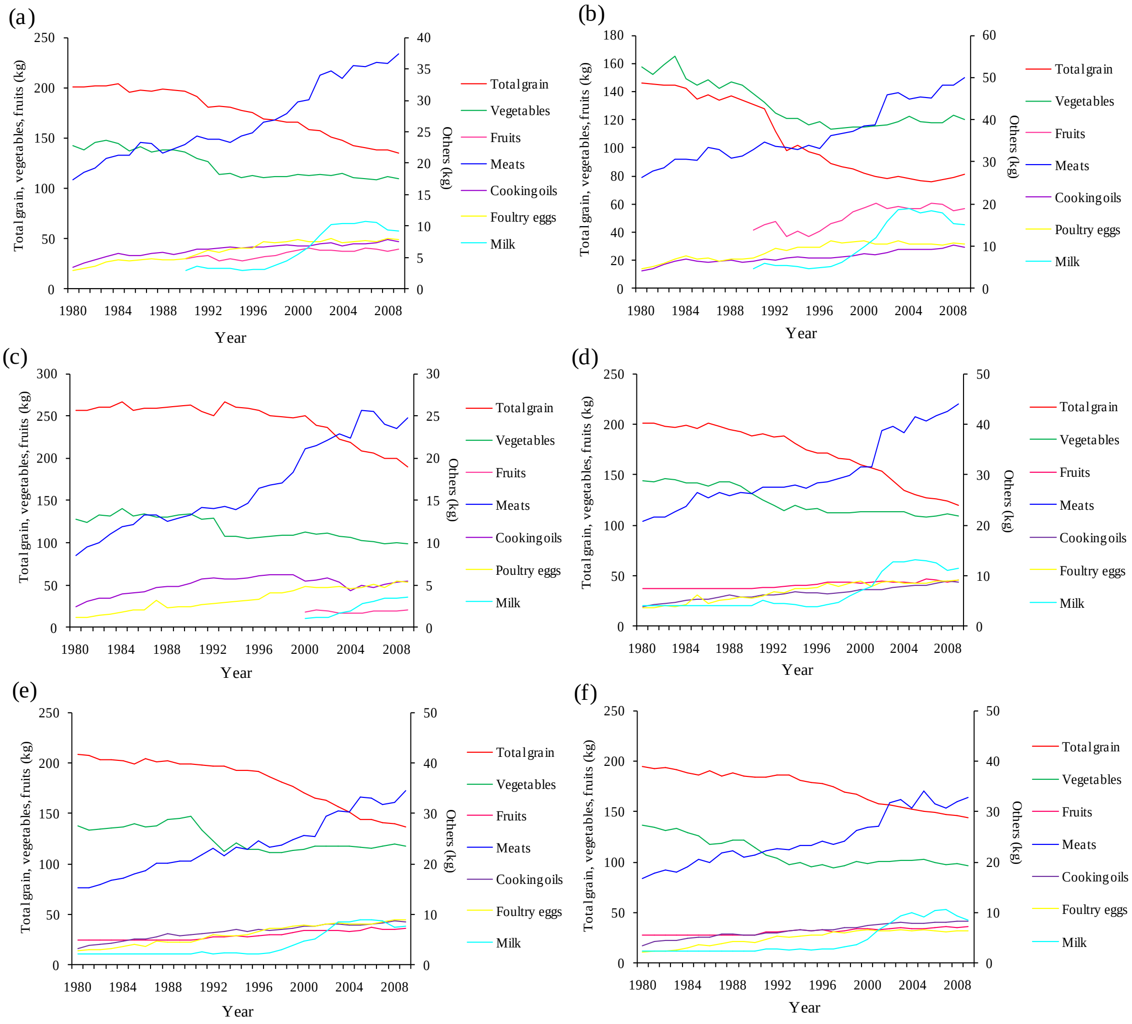

Figure S1 Average annual per capita food consumption in whole China (a), urban (b), rural (c), eastern (d), central (e), and western region (f) from 1980 to 2009. Here the all type of foods virtually indicate unprocessed foods, including domestically produced and imported foods. Per capita food consumptions were estimated by summing domestically and imported unprocessed foods divided by Chinese population.

S2 Administrative provinces and three regions in China

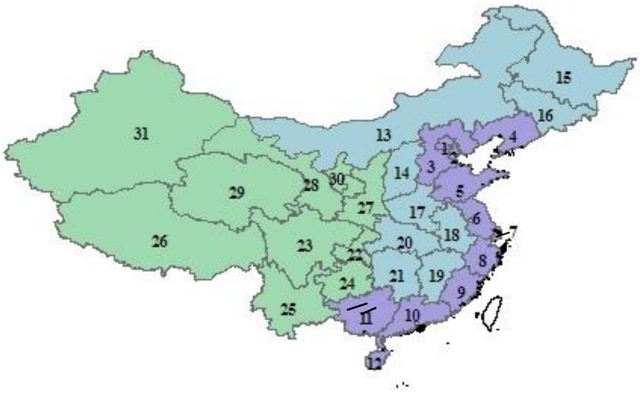

East Region (12 Provinces)		Central Region (9 Provinces)		West Region (10 Provinces)	
1 Beijing	7 Shanghai	13 Inner Mongolia	19 Jiangxi	22 Chongqing	28 Gansu
2 Tianjin	8 Zhejiang	14 Shanxi	20 Hubei	23 Sichuan	29 Qinghai
3 Hebei	9 Fujian	15 Heiloangjiang	21 Hunan	24 Guizhou	30 Ningxia
4 Liaoning	10 Guangdong	16 Jilin		25 Yunan	31 Xinjiang
5 Shandong	11 Guangxi	17 Henan		26 Tibet	
6 Jiangsu	12 Hainan	18 Anhui		27 Shaanxi	

Figure S2 The administrative provinces and three major regions in China. The figure was generated by ArcGIS Desktop (version 10.2, ESRI, Redlands, USA).

In the context of economic and political factors, China was divided into three major regions: East, Central, and West China as shown in **Fig. S2**. With the rapid economic growth, the imbalance of regional economic development become increasingly significant, manifested chiefly by the regional economic disparity of developed eastern coastal China and underdeveloped Midwest. The eastern, central and western regions defined here were divided based on the stair-stepping condition in economic development. The eastern region covers 12 provinces and municipalities, including Beijing, Tianjin, Hebei, Liaoning, Shanghai, Jiangsu, Zhejiang, Fujian, Shandong, Guangdong, Guangxi and Hainan. The central region covers 9 provinces, including Heilongjiang, Jilin, Shanxi, Inner Mongolia, Anhui, Jiangxi, Henan, Hubei and Hunan. The western region covers 10 provinces and municipalities, including Chongqing, Sichuan, Guizhou, Yunnan, Xizang, Shaanxi, Gansu, Qinghai, Ningxia and Xinjiang. Among these three regions, the eastern China region has gained more rapid development historically and concurrently, benefited from favorable climate and geographic conditions, and advanced economy, industries, and agricultures, and hence regarded as the developed area in China. Located in the hinterlands of mainland China, the most western China region is a less developed

area due to poor ecological environments and the lack of sufficient infrastructures. The central China region is inferior to the eastern coastal areas but superior to the western China region in the context of economy, industrialization, advanced agriculture.

S3 Model scenarios

Table S1 Five model scenarios and their relevant explanations

Scenario	Emission features	Dietary patterns
1	Annual changes in emission of 2,3,7,8-TCDD from 1980 to 2009	Annual change in dietary patterns in whole China from 1998 to 2009
2	Fixed emission of 2,3,7,8-TCDD in 1980	Fixed dietary pattern in whole China in 1980
3	Fixed emission of 2,3,7,8-TCDD in 1980	Annual changes in dietary pattern in whole China from 1980 to 2009
4	Fixed emission of 2,3,7,8-TCDD in 1980	Annual changes in dietary pattern in urban residents from 1980 to 2009
5	Fixed emission of 2,3,7,8-TCDD in 1980	Annual changes in dietary pattern in rural residents from 1980 to 2009

S4 Atmospheric 2,3,7,8-TCDD emission in China

Huang et al. established gridded 2,3,7,8-TCDD emission inventory of China with a spatial resolution of 0.25^{o} latitiude $\times 0.25^{o}$ longitude in 2009[5]. According to this method, atmospheric gridded 2,3,7,8-TCDD emission inventory from 1980 to 2008 were established. This inventory was established based on total dioxin emissions in different provinces and different categories in China. However, this emission inventory did not take into consideration of accidental release and release from contaminated soils and water bodies because there were not any ambient measurement data available. Instead, in the model simulations we firstly assumed no concentrations initially in both soils and water bodies. The model was then integrated from 1980 to 2009 with the first year as the model spin-up time, in order to build up soil and water concentrations of 2,3,7,8-TCDD.

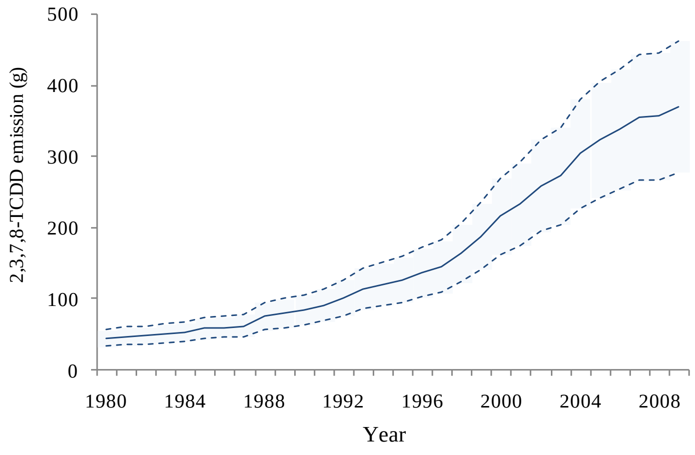

Figure S3 Total atmospheric 2,3,7,8-TCDD emission from 1980 to 2009. The solid line represents 2,3,7,8-TCDD emission using default input parameters and the shaded area represents 95% confidence interval.

S5 CR induced by exposing to 2,3,7,8-TCDD

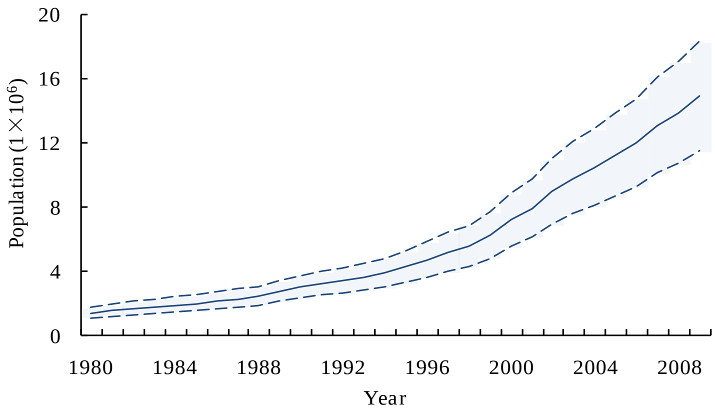

Figure S4 Population with higher cancer risk exceeded acceptable cancer risk of 10^{-4} recommended by the U.S. EPA from 1980 to 2009 derived from the scenario 1 (annual emission of 2,3,7,8-TCDD and dietary patterns from 1980 to 2009). The solid line represents modeled value using default input parameters and the shaded area represent the 95% confidence interval.

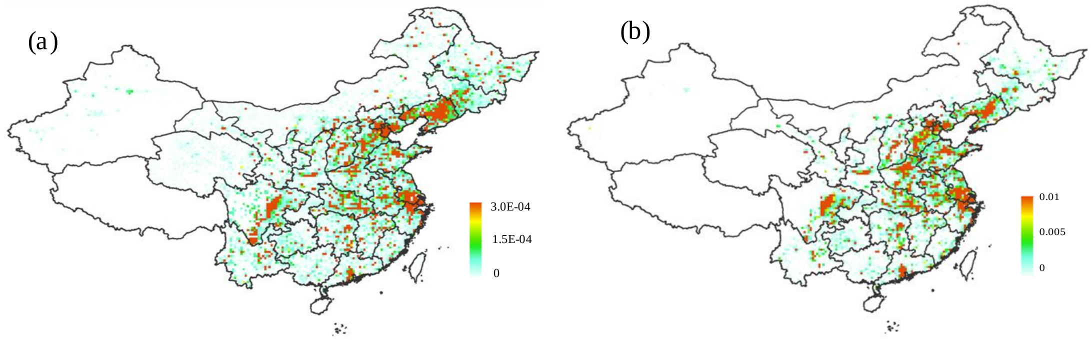

Figure S5 Geographic distribution of annually averaged CR (dimensionless, **a**) and

population-weighted CR (dimensionless, **b**) calculated as products of predicted CR and population density divided by the national average population density in China, induced by exposure to 2,3,7,8-TCDD and simulated using 2,3,7,8-TCDD emission inventory in 2009. The figure was generated by ArcGIS Desktop (version 10.2, ESRI, Redlands, USA).

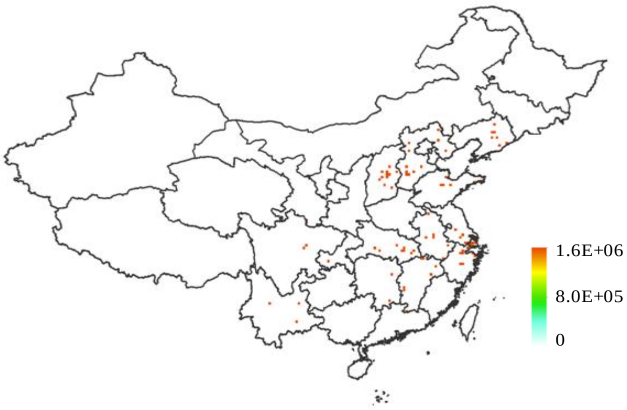

Figure S6 Spatial distribution of population with higher cancer risk exceeded acceptable cancer risk of 10^{-4} recommended by the U.S. EPA in 2009 derived from the model scenario 1 (annual emission of 2,3,7,8-TCDD and dietary patterns from 1980 to 2009, **Table S1**). The figure was generated by ArcGIS Desktop (version 10.2, ESRI, Redlands, USA).

S6 Per capita annual income in China

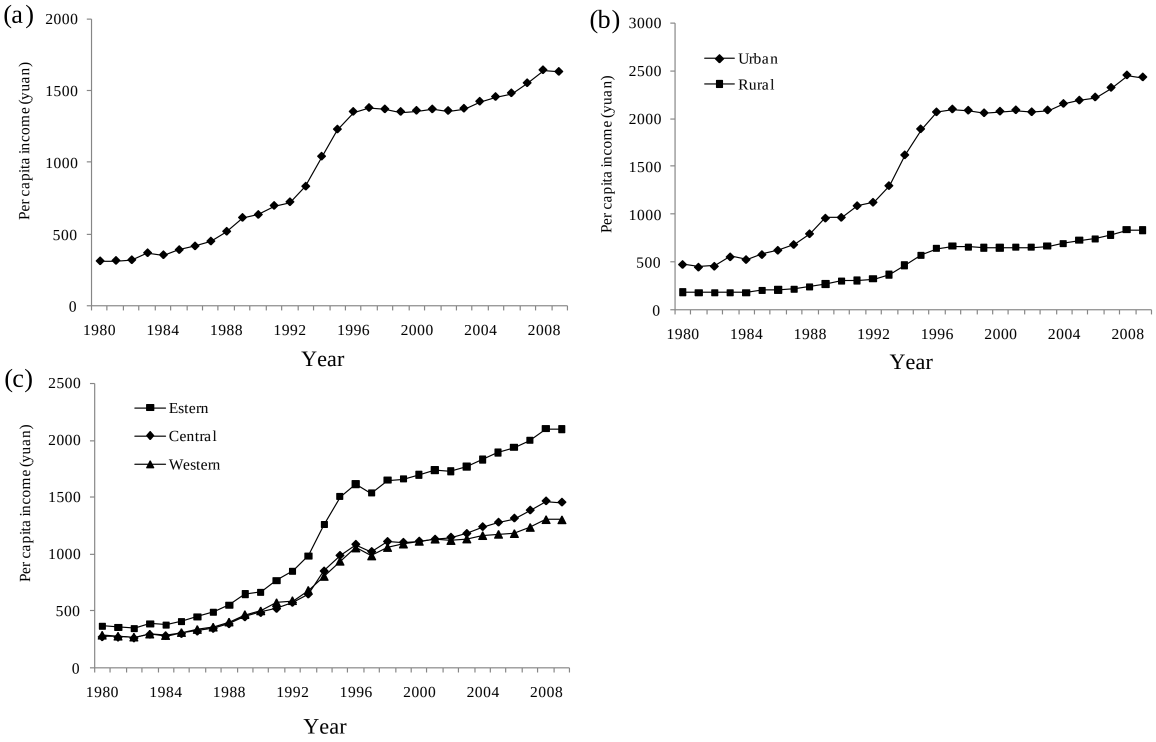

Figure S7 Real annual income per capita calculated based on 1980 price in whole China (a), urban and rural area (b), and different regions (c)

S7 CR and percentage of contribution of various exposure pathways

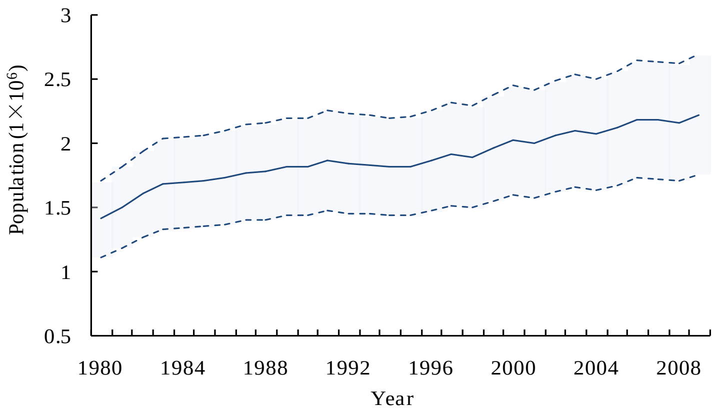

Figure S8 Population with higher cancer risk exceeded acceptable cancer risk of 10^{-4} recommended by the U.S. EPA from 1980 to 2009 derived from the scenario 3 (constant 2,3,7,8-TCDD emission and annual dietary patterns from 1980 to 2009). The solid line represents the modeled cancer risk using default input parameters and shaded area represents the 95% confidence interval.

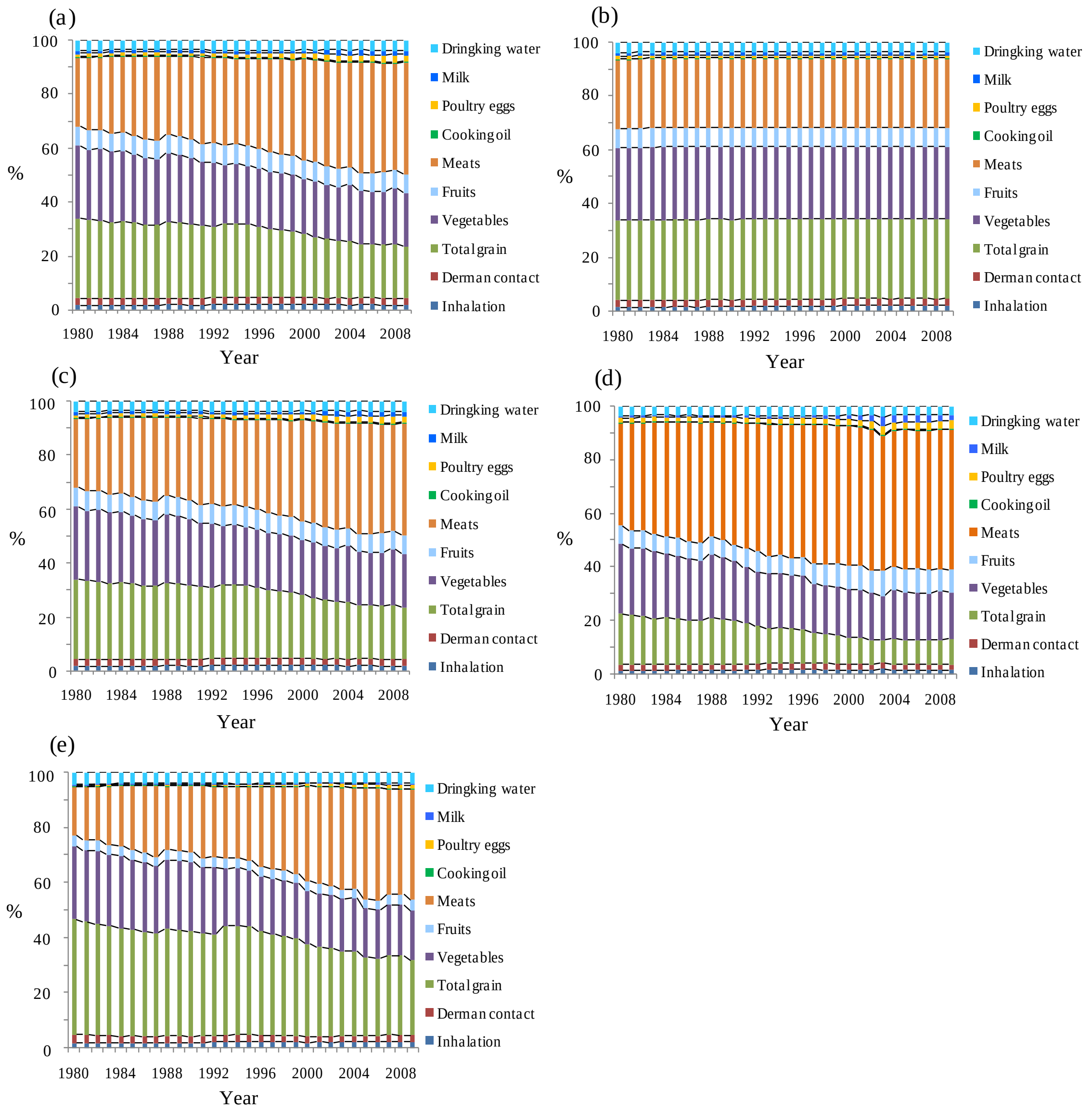

Figure S9 Percentage of contribution of various exposure pathways to the total exposure based on different scenarios. (a) scenario 1 (annual emission of 2,3,7,8-TCDD and dietary patterns from 1980 to 2009); (b) scenario 2 (constant emission of 2,3,7,8-TCDD and dietary patterns in 1980); (c) scenario 3 (constant emission of 2,3,7,8-TCDD in 1980 and variable dietary patterns from 1980 to 2009); (d) scenario 4 (constant emission of 2,3,7,8-TCDD in 1980 and dietary patterns in urban area from 1980 to 2009); (e) scenario 5 (constant emission of 2,3,7,8-TCDD in 1980 and variable dietary patterns in rural area from 1980 to 2009). Exposure pathways include inhalation through air and airborne particles, ingestion of total grain (including all unprocessed grains), vegetables, fruits, meats (pork, beef, mutton, chicken, and fish), milk, cooking oils, poultry eggs, and drinking water, dermal contact with contaminated airborne particles, soil, and water.

S8 CanMETOP Model

S8.1 Model domain and study area

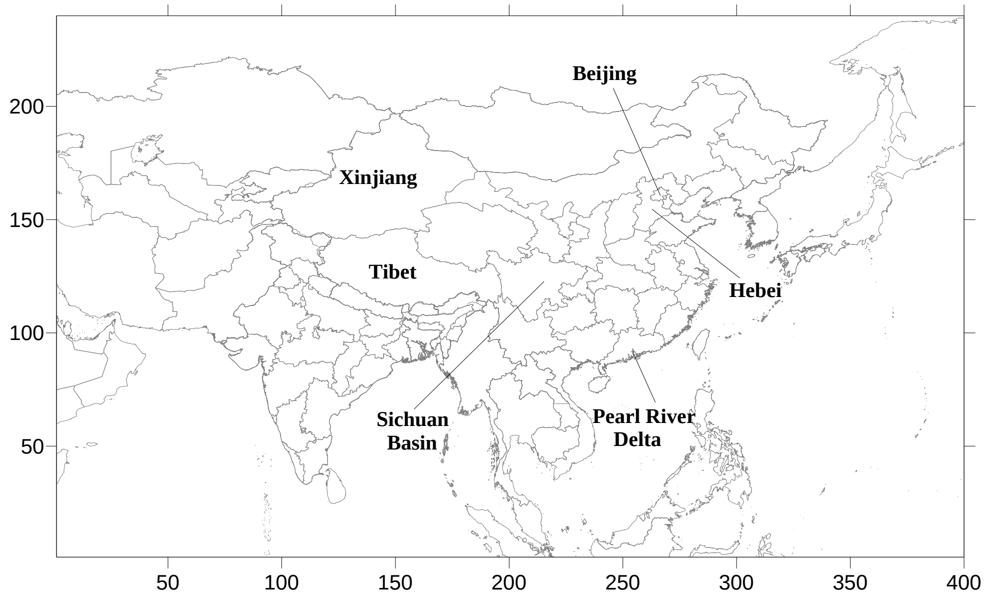

Figure S10 Model domain and study area. The figure was generated by Surfer software (version 8.0, Golden Software, LLC, Colorado, USA).

S8.2 Input parameters

Meteorological data (including winds, atmospheric pressure, temperature, precipitation, etc.) used the 6-hourly objectively analyzed data from the National Centers for Environmental Prediction (NCEP) reanalysis with a spatial resolution of 2.5 °×2.5 ° latitude/longitude[1]. These data were interpolated to the CanMETOP model grids (1/4 °×1/4 °latitude/longitude) and the time step of 20 min. The coarse resolution NCEP winds and temperature data in the surface boundary layer (≤100m) interpolated to the high resolution CanMETOP model grids were adjusted by the Monin-Obukhov similarity theory in the constant flux layer[2]. Geographic data used in the model include terrain height and the surface roughness length. Both data were obtained from the CMC (Canadian Meteorological Centre) which were also interpolated into each model grid. The physicochemical properties of 2,3,7,8-TCDD collected from Mackay et al.[3] were used as the input to CanMETOP and were listed in **Table S2**. The input parameter uncertainties from chemical properties were examined using the default values recommended by MacLeod et al.[4] Based on the gridded atmospheric emission inventory of 2,3,7,8-TCDD in 2009[5], the industrial activities in seven major dioxin-releasing categories from 20 industries were collected, and gridded atmospheric emission inventory of 2,3,7,8-TCDD from 1980 to 2008 were established

and employed in this modeling investigation. The total atmospheric emission of 2,3,7,8-TCDD from 1980 to 2009, and gridded emission of 2,3,7,8-TCDD in 2009 was shown in **Figs. S3** and **S11**, respectively.

Table S2 Physicochemical properties of 2,3,7,8-TCDD used in the modeling study[3]

Parameters	Value	*Cf*	Parameters	Value	*Cf*
Molecular mass (g/mol)	321.97	1	Water solubility (g/m^3)	0.0002	1.5
Molar volume (cm^3/mol)	188.34	1	Log K_{ow}*	2.78+282.2/T (K)	1.1
Melting point (^{o}C)	295.0	1	BCF*	38.73$K_{ow}^{-0.578}$	1.5†
Entropy of Fusion (J/mol K)	69	1	Log K_{oc}*	5.67	1.5†
Degradation rate in air (%)	9.67×10^{-5}	2	Log H* (Pa m^3/mol)	9.94-3117/T (K)	1.5†
Degradation rate in soil (%)	1.14×10^{-6}	2	BCF_m	4.32	1.5†
Liquid vapor pressure (Pa)	3.35×10^{-4}	1.5			

*: K_{ow} and K_{oc} are octanol-water and water-organic partition coefficient, respectively. BCF is bioconcentration factors and BCF_m is meat and milk fat bioconcentration factors[6]. H is Henry's law constant. K_{ow}, BCF and H are temperature dependent. †: *Cf* (confidence factor) in this study due to the lack of data.

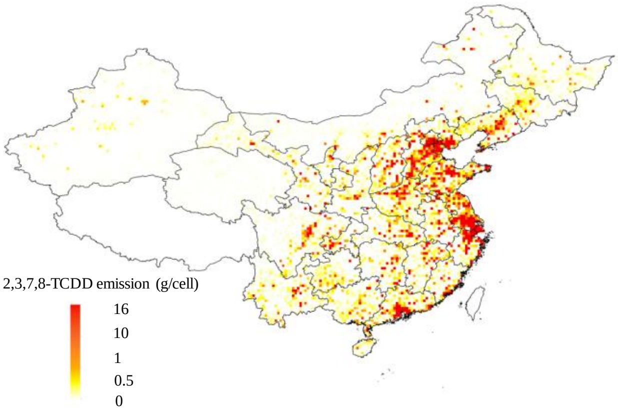

Figure S11 Distribution of 2,3,7,8-TCDD emission in China in 2009 with a 1/4° longitude by 1/4° latitude resolution. The figure was generated by ArcGIS Desktop (version 10.2, ESRI, Redlands, USA).

S8.3 Model evaluation

The CanMETOP has been extensively evaluated and verified against available monitored air concentration data collected from the globe[2,7-10]. To further establish the level of confidence in the present model investigation, further efforts were made to evaluate and verify modeling results against monitored concentration of 2,3,7,8-TCDD across China. There have been no extensive measurement data available in China and worldwide due to high cost in sampling and

laboratory analysis, particularly the routine measurement data. In China, most field studies on dioxins atmospheric levels have been carried out in individual megapolis or the area near emission sources. Vegetation, a commonly used passive air sampler, has been employed as a bio-indicator for atmospheric contamination levels of lipophilic compounds, such as polycyclic aromatic hydrocarbons (PAHs), polychlorinated biphenyls (PCBs), and PCDD/Fs[11-15]. Chen et al.[16] have established a relationship between PCDD/Fs concentrations in pine needles and air from their field sampling in 38 Cities across China. To apply these data in the evaluation of the modeled air concentrations, five air samples of 2,3,7,8-TCDD collected from Beijing, Tianjin, Shanghai, Wuhan, and Guangzhou were compared with 2,3,7,8-TCDD concentrations (pg/g dw) in pine needles. Details of these data were listed in **Table S3**. The relationship between 2,3,7,8-TCDD concentrations in pine needles and ambient air concentrations is illustrated in **Fig. S12**. These two datasets show an excellent correlation (*r*=0.99, p<0.001), suggesting the feasibility of using pine needles-derived concentrations in the verification of the modeled data.

Table S3 2,3,7,8-TCDD levels in air and pine needles

Site	Air				Pine needles			
	Date Year. mon. data	Sample size	Measured pg/m^3	Ref.	Date Year. mon. data	Sample size	Measured pg/g dw	Ref.
Beijing	08.01	5	0.007	Sun, 2009	09.09-09-10	4	0.13	16
Tianjin	09.01.01-09.01.05	4	0.0169	Ding et al., 2012	09.09-09-10	4	0.29	16
Shanghai	09.03	3	0.02	Chen et al., 2011	09.09-09-10	5	0.33	16
Wuhan	09.09-09-10	4	0.01	Chen et al., 2012	09.09-09-10	4	0.14	16
Guangzhou	09.09-09-10	5	0.03	Chen et al., 2012	09.09-09-10	5	0.53	16

ND: Not detected

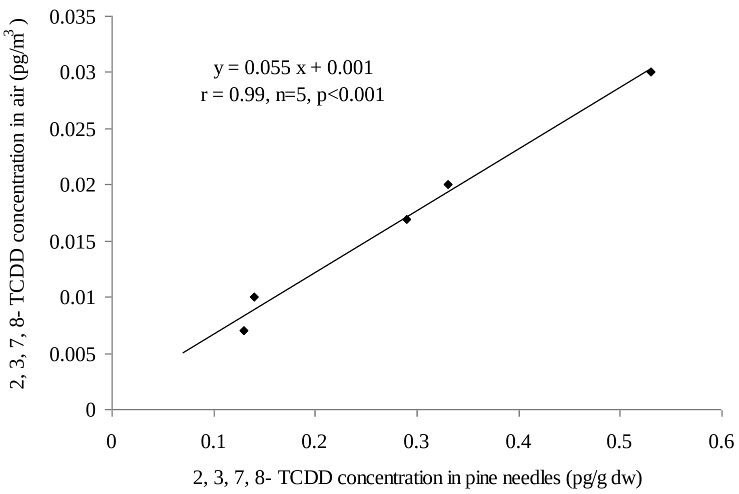

Figure S12 Relationship between 2,3,7,8-TCDD concentrations (pg/g dw) in pine needles and in air samples (pg/m^3). n is the number of data points used in correlation analysis, r is the correlation coefficient, and p is the significance level.

As aforementioned, most measured dioxins air concentrations were collected at a single or a few sites within urban centers where dioxins concentrations were expected to be higher than that in surrounding suburban and rural areas, or vice versa. It should be noted that the predicted 2,3,7,8-TCDD concentration at a model grid with grid spacing of 27.83 ×27.83 km^2 (1/4 °×1/4 ° latitude/longitude) stands for the mean concentration over the grid cell which often includes both urban and rural areas. In addition, in the 38 cities, Liyang exhibited the highest 2,3,7,8-TCDD concentrations in pine needle (0.94 pg/g dw). This value exceeded four standard deviation (0.17) of 38 samples data series and hence was omitted from the pine needles dataset. Since monitored 2,3,7,8-TCDD air concentrations in three cities (Beijing, Tianjin, and Shanghai) included in the pine needled dataset were available in literature[17-19], we also replaced the pine needle data in these three cities by the monitored ambient air concentrations. Thus, pine needles sampled 2,3,7,8-TCDD concentrations in the rest 34 cities[16] were employed for model validation. These pine needles data were converted to air concentrations using the linear relationship presented in **Fig. S12**. In addition, ambient 2,3,7,8-TCDD concentrations at 20 sites collected from literature[20-25] were also used to compared with modeled concentrations. **Table S4** presents monitored data from pine needles and ambient measurements, modeled air concentrations, and error quotients between measured and modeled data.

Large spatial variability in the measured 2,3,7,8-TCDD concentrations might also contribute

to the differences between the predicted and measured 2,3,7,8-TCDD concentrations. For example, measured 2,3,7,8-TCDD air concentrations ranged from 0.009 to 0.026 pg/m^3 at different sites over an area of 12 km^2 in Tianjin, a mega city near Beijing.[18] Because measured 2,3,7,8-TCDD air concentrations were reported at only limited number of sites in big cities and collected during a short period of time, their deviations from modeled daily concentrations were expected. **Figure S13(a)** displays the sampling sites in those field campaigns. The data collected from these sites were used to compare with modeled concentrations. Results are presented in **Fig. S13(b)**. As seen, the modeled air concentrations at the second model level (1.5 m above the surface) match very well with the measured data at a correlation coefficient of r =0.79 ($p<0.001$) and low values of error quotients (EQs, **Table S4**).

We further compared modeled 2,3,7,8-TCDD concentrations in soil with measured soil concentrations (ng/g, **Table S5**). Result shows that the EQs between modeled and measured soil data range from 0.23 to 6.4. It is worthwhile to note that most soil samples were collected near dioxin emission sources (point or area sources), such as electronic waste recycling sites (Taizhou and Guiyu), where higher levels of 2,3,7,8-TCDD were always observed. The model underestimated levels of this toxic chemical in soil near these sampling sites. This is because the modeled gridded soil concentrations were virtually the mean concentrations averaged over a grid cell (1/4 °×1/4 °latitude/longtitude) which could not pinpoint a single monitoring site. This would inevitably lead to the difference between area-averaged concentrations and site-specific concentrations. Nevertheless, at those model grids far away from major dioxin emission sources and contaminated sites, modeled soil concentrations were higher than measured concentrations, such as Dalian, Changshan, and Shanghai (**Table S5**).

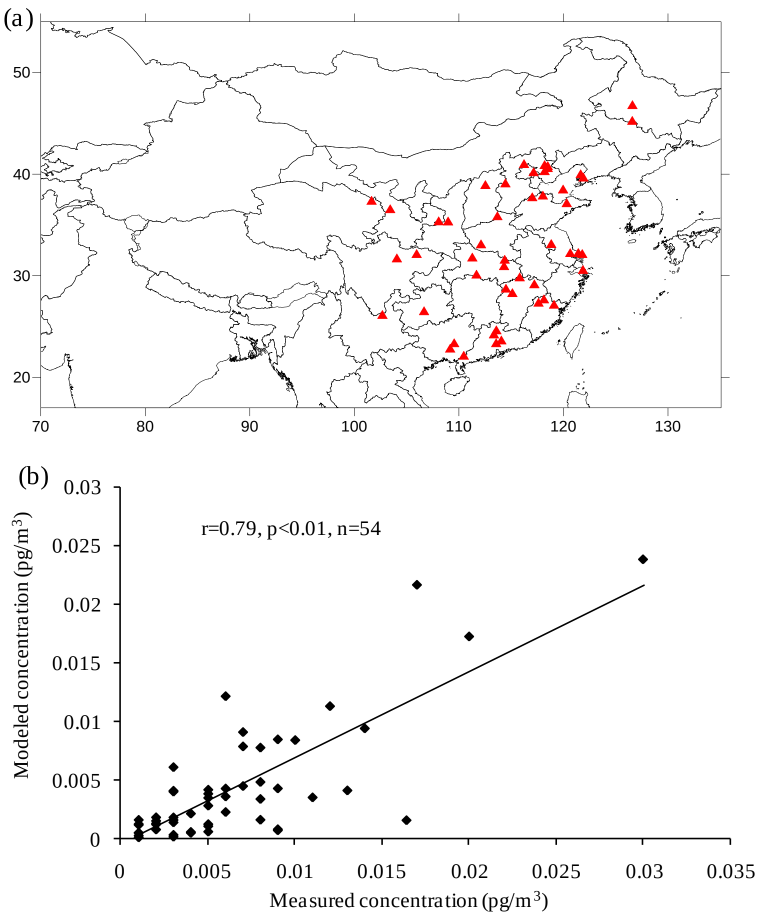

Figure S13 Comparison between modeled and sampled air concentrations of 2,3,7,8-TCDD acrcoss China. **(a)** Sampling sites selected from different field campaigns by different research groups; **(b)** correlation diagram for modeled and monitored air concentrations (pg/m^3). *n* is the number of data points used in correlation analysis, *r* is the correlation coefficient, and *p* is the significant level. The figure (a) was generated by ArcGIS Desktop (version 10.2, ESRI, Redlands, USA).

Table S4 Comparison of the modeled and measured air concentrations (pg/m^3) of 2,3,7,8-TCDD and calculated EQ values at all monitoring sites.

Site	Sample	Ref.	Date	Sample size	Method[1]	Measured concentration		Modeled (pg/m^3)	EQ [c]
						Pine needles (pg/g dw)	Atmosphere (pg/m^3)		
Beijing	TSP	20	04.04	24	Hiv, HRGC-HRMS		0.007	0.0079	1.1
Beijing	PM10	17	08.01	5	Mev, HRGC-HRMS		0.007	0.004526	0.6
Tianjin	TSP	18	09.01.01-09.01.05	5	Hiv, HRGC-HRMS		0.017	0.02165	1.3
Shanghai	TSP	21	08.03	3	Hiv, HRGC-HRMS		0.002	0.001868	0.9
Shanghai	TSP	21	09.03	1	Hiv, HRGC-HRMS		0.006	0.004318	0.7
Shanghai	TSP	19	09.03	3	Hiv, HRGC-HRMS		0.020	0.017257	0.9
Shanghai	TSP	21	09.03	1	Hiv, HRGC-HRMS		0.003	0.00186	0.6
Shanghai	TSP	21	09.03	2	Hiv, HRGC-HRMS		0.006	0.003642	0.6
Wuhan	TSP	16	09.09-09.10	1	Hiv, GC-HRMS		0.010	0.008438	0.8

Guangzhou	TSP	22	04.07-04.09	4	Hiv, HRGC-HRMS		0.005	0.004216	0.8
Guangzhou	TSP	16	09.09-09.10	1	Hiv, GC-HRMS		0.030	0.02382	0.8
Dalian	TSP	23	09.12.15-09.12.23	1	Hiv, GC-HRMS		0.001	0.00165	1.7
Dalian	TSP	23	09.12.15-09.12.23	1	Hiv, GC-HRMS		0.002	0.001348	0.7
Tangshan	PM10	24	09.06.16-09.06.26	3	Hiv, GC-HRMS		0.003	0.006135	2.0
Tangshan	PM10	24	09.06.16-09.06.26	3	Hiv, GC-HRMS		0.007	0.009121	1.3
Tangshan	PM10	24	09.06.16-09.06.26	3	Hiv, GC-HRMS		0.006	0.012173	2.0
Tangshan	PM10	24	09.06.16-09.06.26	3	Hiv, GC-HRMS		0.014	0.009436	0.7
Shenzhen	TSP	25	09.02-09.3	6	Hiv, HRGC-HRMS		0.009	0.0085	0.9
Xiangshan	TSP	19	09.03	3	Hiv, HRGC-HRMS		0.008	0.0078	1.0
Fuzhou	TSP	19	09.07	6	Hiv, HRGC-HRMS		0.005	0.003526	0.7
Xianning	TSP	19	09.07	3	Hiv, HRGC-HRMS		0.013	0.004156	0.3
Nanchong	TSP	19	09.08	2	Hiv, HRGC-HRMS		0.003	0.004054	1.4
Harbin	Pine needles	16	09.09-09.10	4	GC-HRMS	0.23	0.012[b]	0.011326	0.9
Jilin	Pine needles	16	09.09-09.10	4	GC-HRMS	0.07	0.003[b]	0.000215	0.1
Shijiazhuang	Pine needles	16	09.09-09.10	5	GC-HRMS	0.16	0.008[b]	0.003428	0.4
Taiyuan	Pine needles	16	09.09-09.10	4	GC-HRMS	0.18	0.009[b]	0.004328	0.5
Laizhou	Pine needles	16	09.09-09.10	5	GC-HRMS	0.04	0.001[b]	0.000543	0.5
Zibo	Pine needles	16	09.09-09.10	4	GC-HRMS	0.07	0.003[b]	0.004129	1.4
Jinan	Pine needles	16	09.09-09.10	4	GC-HRMS	0.12	0.006[b]	0.002316	0.4
Xining	Pine needles	16	09.09-09.10	3	GC-HRMS	0.06	0.002[b]	0.00083	0.4
Qingdao	Pine needles	16	09.09-09.10	5	GC-HRMS	0.00	0.001[b]	0.00032	0.3
Lanzhou	Pine needles	16	09.09-09.10	5	GC-HRMS	0.28	0.0164	0.00162	0.1
Zhengzhou	Pine needles	16	09.09-09.10	4	GC-HRMS	0.06	0.004[b]	0.002187	0.5
Yangling	Pine needles	16	09.09-09.10	5	GC-HRMS	0.14	0.009[b]	0.000853	0.1
Xi’an	Pine needles	16	09.09-09.10	5	GC-HRMS	0.03	0.003[b]	0.001645	0.5
Nanjing	Pine needles	16	09.09-09.10	5	GC-HRMS	0.18	0.011[b]	0.003563	0.3
Xiangyang	Pine needles	16	09.09-09.10	5	GC-HRMS	0.02	0.002[b]	0.001568	0.8
Wujiang	Pine needles	16	09.09-09.10	4	GC-HRMS	0.00	0.001	0.0013	1.3
Chengdu	Pine needles	16	09.09-09.10	5	GC-HRMS	0.08	0.005[b]	0.00286	0.6
Yichang	Pine needles	16	09.09-09.10	3	GC-HRMS	0.07	0.005[b]	0.00065	0.1
Changde	Pine needles	16	09.09-09.10	4	GC-HRMS	0.12	0.008[b]	0.001659	0.2
Nanchang	Pine needles	16	09.09-09.10	5	GC-HRMS	0.13	0.008[b]	0.004875	0.6
Guixi	Pine needles	16	09.09-09.10	4	GC-HRMS	0.02	0.002[b]	0.001264	0.6
Yichun	Pine needles	16	09.09-09.10	4	GC-HRMS	0.03	0.003[b]	0.001438	0.5
Ji’an	Pine needles	16	09.09-09.10	3	GC-HRMS	0.05	0.004[b]	0.000623	0.2
Nanping	Pine needles	16	09.09-09.10	3	GC-HRMS	0.03	0.003[b]	0.000376	0.1
Sanming	Pine needles	16	09.09-09.10	4	GC-HRMS	0.00	0.001[b]	0.00056	0.6
Guiyang	Pine needles	16	09.09-09.10	4	GC-HRMS	0.07	0.005[b]	0.001287	0.3
Kunming	Pine needles	16	09.09-09.10	3	GC-HRMS	0.05	0.004[b]	0.000549	0.1
Conghua	Pine needles	16	09.09-09.10	4	GC-HRMS	0.07	0.005[b]	0.003874	0.8
Zhuhai	Pine needles	16	09.09-09.10	4	GC-HRMS	0.14	0.009[b]	0.000763	0.1
Pubei	Pine needles	16	09.09-09.10	3	GC-HRMS	0.00	0.001[b]	0.001225	1.2
Zhanjiang	Pine needles	16	09.09-09.10	4	GC-HRMS	0.07	0.005[b]	0.001115	0.2

Ledong	Pine needles	16	09.09-09.10	3	GC-HRMS	0.0	0.001[b]	0.00016	0.2

[1]HiV: High volume sampler (~1 m^3/min), MeV: Medium volume sampler (~20 L/min) ; [b] calculated from 2,3,7,8-TCDD concentrations in pine needles; [c] EQ: error quotient, defined as the ratio between the modeled concentrations and the observed data.

Table S5 Comparison between modeled and measured 2,3,7,8-TCDD concentrations (ng/g) in soil and calculated EQ values at all monitoring sites.

Site	Ref.	Data	Sample size	Method[1]	Measured concentration	Modeled (ng/g)	EQ[1]
Shanghai	21	2009.06.07	4	HRGC-HRMS	0.293	1.126	3.84
Dalian	26	2002.12	2	GC-HRMS	0.110	0.903	8.21
Wolong	27	2006.08	5	HRGC-HRMS	0.114	0.058	0.51
Yangtze River delta	28	2004.06	2	HRGC-HRMS	1.560	1.048	0.67
Jinhua	29	2008	3	GC-HRMS	0.800	0.186	0.23
Jiaxing	29	2008	3	GC-HRMS	2.600	1.103	0.42
Taizhou	29	2008	3	GC-HRMS	0.800	0.246	0.31
Yongjia	29	2008	3	GC-HRMS	0.200	0.378	1.89
Changshan	29	2008	3	GC-HRMS	0.100	0.64	6.40
Guiyu	30	2004.02	3	HRGC-HRMS	7.040	5.983	0.85
Baiyin	31	2008.09	3	HRGC-HRMS	0.025	0.052	2.08

[1]Error quotient, defined as the ratio between the modeled concentrations and the observed.

S9 Food web model

2,3,7,8-TCDD contamination to the web food of Chinese residents can be estimated using a food chain model. The food chain model inputs CanMETOP modeled 2,3,7,8-TCDD concentrations in air and airborne particles at the 1.5 m height (the first model level above the grounded surface), soil and water (**Fig. S14**).

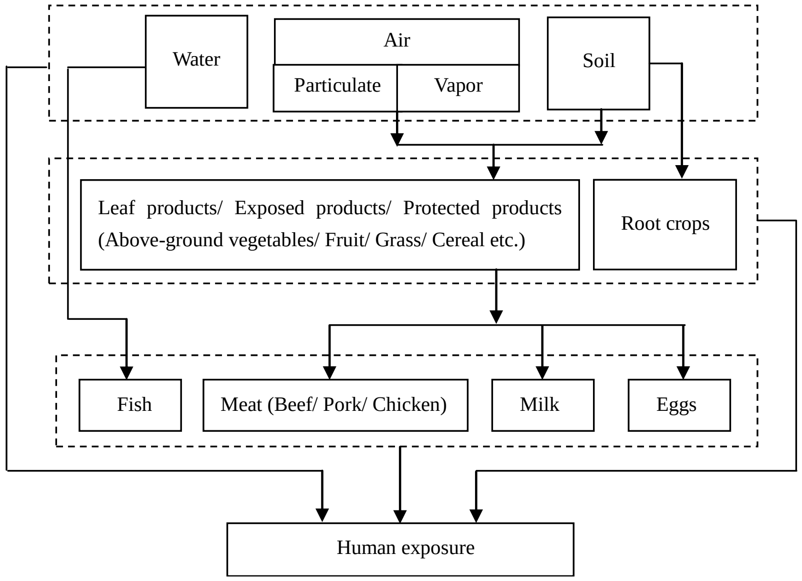

Figure S14 Flow chart showing input of 2,3,7,8-TCDD from different environmental compartments to food web and major contamination routes in food chain

S9.1 TCDD concentrations in botanic-food

2,3,7,8-TCDD accumulation in a plant may occur through several routs: (1) dry gaseous atmospheric deposition (through air-soil or air-plant exchange processes), (2) wet and dry particle atmospheric deposition, and (3) root uptake from the soil and translocation within the plant:[6]

$$C_g = (C_v + C_p + C_r) \times d_w \quad (1)$$

where C_g is the overall concentration in plant (pg/kg dry weight if d_w is omitted, wet weight if d_w is included). C_r is the concentration in plant due to root uptake (pg/kg dw). C_v is the concentration in plant due to dry gaseous deposition (pg/kg dw). C_p is the concentration in plant due to wet and dry particle atmospheric deposition. d_w is a dry-to-wet weight conversion factor [(mg/kg ww)/(mg/kg dw)] (**Table S6**). Contaminant mass fractions in homegrown product consumed by human, chicken, and pig were calculated in terms of wet weight. Feed consumption by cattle was reported on a dry-weight basis[6]. However, all cereals were estimated on a dry-weight basis.

Theoretically, the mass fraction of a contaminant in each type of botanic-food can be evaluated individually. A paucity of empirical data, however, necessitates the categorization of vegetables, fruits, and cereals into a limited number of groups. Based on available data in

literature[32] and the guidance from New York State Department of Health[33], products grown for human consumption can be divided into three categories: leafy product, exposed product, and protected product. Leafy product (e.g. grass, spinach and lettuce) is characterized by the relatively large interception fractions of their edible portion, which suggests that concentration in plant from direct deposition should be of greater importance relative to non-leafy vegetation. Exposed products (e.g. tomatoes, bell peppers, and strawberries) include non-leafy fruits and vegetables for which the edible portion is grown above the ground. Last, protected products (e.g. oranges, bananas, and corn) include botanic-food which is not exposed to direct deposition, and the edible portion is protected by an inedible casing.

Table S6 Botanic-food modeling parameters

	above-ground crop			References
	Leaf	Exposed	Protected	
Root uptake bioconcentration factor for crop (BCF_r)	0.013	0.013	0.013	6
Mass based air-to-plant foliage transfer factor due to dry gaseous deposition (B_{vpa})	1.43×10^5	1.43×10^5	1.43×10^5	6
Fraction of total air concentration present in vapour phase (f_v)	0.5	0.1	0.01	6
Fraction of wet deposition adhering to plant (F_w)	0.3	0.3	0.3	6
Fraction of particles intercepted by plant (R_p)	0.1192	0.0319	NA[a]	33
Plant surface particle loss rate constant (k_p, yr^{-1})	18	18	NA[a]	34
Duration of plant exposure. Due to deposition per harvest (T_p, yr)	0.42	0.42	NA[a]	34
Yield (Y_p, kg dw/m^2)	0.099	0.126	0.222	34
Dry-to-wet weight conversion factor (d_w)	0.066	0.126	0.222	34
Correction factor reflecting the difference between vegetable and the vegetable oil (VG_v)		0.1		6

[a]NA: not available. The values of exposed plant were adopted to estimate contaminate bio-concentration in protected plant.

Contaminant mass fraction in botanic-food due to root uptake is given by:

$$C_r = C_s \times BCF_r \tag{2}$$

where C_s is the concentrations in soil (pg/kg). BCF_r is a root uptake bioconcentration factor for crop (**Table S6**).

The dry gaseous atmospheric deposition is estimated by

$$C_v = (B_{vpa} \times f_v \times C_a) / d_a \quad (3)$$

where B_{vpa} is mass based air-to-plant foliage transfer factor due to dry gaseous deposition. f_v is an adjustment factor accounting for the tendency for contaminates to remain in the exposed surfaces of vegetation. d_a is the density of air (1.19 kg/m^3 at standard conditions). These parameters are listed in **Table S6**.

The wet and dry particle deposition is determined as follows:[6]

$$C_p = (D_{yd} + (F_w \times D_{yw})) \times R_p \times (1 - \exp(-k_p \times T_p)) / (Y_p \times k_p) \quad (4)$$

where D_{yd} is annual dry particle deposition flux (pg/m^2/yr). F_w is fraction of wet deposition adhering to plant. D_{yw} is annual wet particle deposition flux (pg/m^2/yr). R_p is fraction of particles intercepted by plant. k_p is the effective removal (or degradation) rate from plant surfaces. T_p is the length of the growing season (yr). Y_p is the crop yield (kg/m^2). D_{yd} and D_{yw} were obtained from the output of CanMETOP, and other parameters are listed in **Table S6**.

For the root crop (e.g. potatoes and carrots), the root-zone uptake is the main entry pathwa, and the contaminate concentration can be estimated using Equation (2) [35].

Due to unavailability of data, it was assumed that four categories: leafy product, exposed product, protected product, and root products respectively accounted for 1/4 of the total consumption of vegetables. Fruits mainly was composed of exposed product and protected product, and each of them accounted for half of the total fruits.

For vegetable oil, it was assumed that they were mainly produced from crops characterized with protected product, and the concentration in vegetable oil can be estimated as follows:

$$C_{vo} = C_g \times VG_v \quad (5)$$

where C_{vo} is concentration in vegetable oil. VG_v is a correction factor reflecting the difference between vegetable and the vegetable oil (**Table S6**).

S9.2 Estimation of concentrations in terrestrial animal-derived foodstuffs

Pork, beef, mutton, and chicken as prevailing raw meats from animals for Chinese were considered as meat in the model. Here, the concentration of a pollutant in an animal tissue, milk or egg is directly related to its concentration in the animal's diet, using a bioconcentration factor, defined by:

$$C_x = BCF_x \times C_d \quad (6)$$

where C_x is the concentration in animal foodstuff x (pg/kg). BCF_x is a fresh weight bioconcentration factor of animal foodstuff *x*. C_d is the concentration in diet of animal from which foodstuff *x* is derived (pg/kg).

BCF_x values for meat and milk were modeled by assuming that they were identical to BCF on a fat weight basis. BCF_x can be calculated by

$$BCF_x = BCF_m \times X_{ff} \quad (7)$$

where X_{ff} is fat fraction of meat and milk (**Table S7**). BCF_m is a fat concentration factor of 2,3,7,8-TCDD (4.32) [3].

Bioconcentration factor of eggs can be estimated by

$$BCF_e = BCF_m \times X_{ef} \quad (8)$$

where BCF_e is a fresh weight egg yolk bioconcentration factor. X_{ef} is fat fraction of egg yolk (**Table S8**).

Food ingestion pathway has been identified as the major pathway of human dioxin intake in the previous studies[36,37]. Analogous to human, in this model, chickens, eggs and meat, and livestock (cattle and pig), meat and milk were also assumed to come from three sources only: grass / vegetables, fodder/grain and soil.

C_d for each animal was calculated using the animal-specific values of F_f, F_g, and F_s as listed in **Table 8**.

$$C_d = F_g \times C_g + F_f \times C_f + F_w \times C_w + F_s \times C_s \quad (9)$$

where F_g, F_f, F_w, and F_s, are the fraction of animals' diet, namely grass/vegetables, fodder/grain, water and soil, respectively (**Table S8**).

Table S7 Density and lipid content of food[38]

	Density (kg/m^3)			Lipid(g/kg)		
	Default value	*CV* (%)	*Cf*	Default value	*CV* (%)	*Cf*
Beef	1400	8	1.2	24	24	1.6
Pork	975	13	1.3	370	32	1.9
Chicken	1200	10	1.2	94	16	1.4

Eggs	1085	8	1.2	88	9	1.2
Fish	1450	18	1.4	9.1	28	1.8
Milk	1028.8	9	1.2	32	11	1.2

Table S8 Dietary patterns of animals

	F_g			F_f	F_s	References
	Leaf	Exposed	Protected			
Pig	0.10	0.10	0.10	0.66	0.04	assumptions based on feedlot fattening pigs[6], but allowing for additional vegetables ingestion by traditional farm pigs.
Beef cattle	0.48			0.48	0.04	6
Milk cattle	0.96			0	0.04	6
Sheep	0.96				0.04	assumptions based on beef cattle
Chicken	0.10			0.86	0.04	6

S9.3 2,3,7,8-TCDD concentrations in fish

2,3,7,8-TCDD concentration in fish body can be assessed by assuming the equilibrium of the fugacity in fish and water[39].

$$C_f = \frac{C_w \times BCF \times O_f}{den_f} \tag{2}$$

where C_f is 2,3,7,8-TCDD concentration in fish. BCF is a bio-concentration factor of 2,3,7,8-TCDD (18.87) [3]. O_f and den_f are lipid content and density of fish, respectively (**Table S6**).

For input parameters with unavailable CVs in the food web model, the Cf_s was assumed to be 1.5 and were used to uncertainty analysis.

S9.4 Food wed model evaluation

To evaluate the web model performance, measured 2,3,7,8-TCDD concentration in the diet were collected to compare with modeling results across China. Unfortunately, measurement data available were very scare and they were mainly concentrated in fish in China. To evaluate the modeled concentrations, measured 2,3,7,8-TCDD concentration in fish, chicken, and egg were collected from literatures[40-49]. **Figure S15** displays the spatial pattern of the sampling sites in these studies. Details of these data and error quotients between modeled and measured data were listed in **Table S9**. The error quotients ranged from 0.18 to 6.13 (**Table S9**), indicating that

modeled and measured 2,3,7,8-TCDD concentration have the same order of magnitude. It should be noted that most samples were collected at a single or a few sites, however, the predicted 2,3,7,8-TCDD concentration in the food at a model grid with grid spacing of 27.83 ×27.83 km^2 (1/4 °×1/4 ° latitude/longitude) stands for the mean concentration over this grid cell. In addition, 2,3,7,8-TCDD concentration levels in various environmental media from 1980 to 2009 were predicted based on a high-resolution emission inventory in 2009[5]. All these factors might contribute to the differences between the predicted and measured 2, 3, 7 ,8-TCDD concentrations.

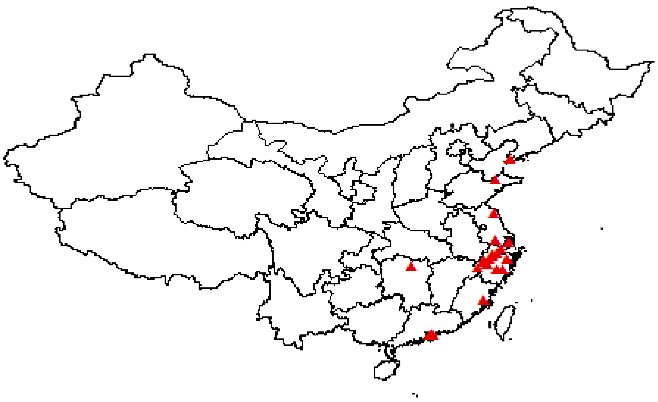

Figure S15 Sampling sites selected by different research groups. The figure was generated by ArcGIS Desktop (version 10.2, ESRI, Redlands, USA).

Table S9 Comparison between modeled and measured 2,3,7,8-TCDD concentrations (pg/g) in food and calculated EQ values at all monitoring sites

Site	Ref.	Sample	Data	Sample size	Measured concentration (pg/g)	Modeled concentration (pg/g)	EQ [a]
Shenzhen	40	fish	2003	1	0.180	0.126	0.699
Shenzhen	41	fish	2003	20	0.046	0.042	0.913
Shenzhen	42	fish	2008	16	0.086	0.062	0.724
Jinhua	43	fish	2005	2	0.06	0.039	0.649
Hangzhou	43	fish	2005	1	0.069	0.036	0.524
Hangzhou	43	fish	2005	1	0.024	0.036	1.483
Jiaxing	43	fish	2005	2	0.058	0.068	1.178
Quzhou	43	fish	2005	2	0.033	0.029	0.877
Pearl river delta	44	fish	2006	31	0.031	0.13	4.2
Dongting lake	45	fish	2004	17	0.058	0.033	0.567
Lanxi	46	fish	2009	8	0.13	0.025	0.19
Jiande	46	fish	2009	5	0.02	0.014	0.721
Tonglu	46	fish	2009	6	0.038	0.025	0.667
Fuyang	46	fish	2009	7	0.29	0.185	0.638

Xiaoshan	46	fish	2009	4	0.31	0.149	0.48
Yongjia	46	fish	2009	1	0.01	0.049	4.926
Taizhou	46	fish	2009	1	0.35	0.244	0.696
Jiaxing	46	fish	2009	1	0.04	0.074	1.86
Changshan	46	fish	2009	1	0.02	0.014	0.721
Dalian	47	fish	2008	1	0.07	0.074	1.054
Laizhou	47	fish	2008	1	0.05	0.03	0.606
Yancheng	47	fish	2008	1	0.015	0.017	1.144
Ningbo	47	fish	2008	1	0.015	0.044	2.94
Fuzhou	47	fish	2008	1	0.02	0.069	3.436
Wuxi	48	fish	2002	6	0.09	0.193	2.141
Shenzhen	49	Chicken	2006	13	0.0018	0.011	6.227
Shenzhen	49	Egg	2006	13	0.0038	0.01	2.724

[a] error quotient, defined as the ratio between the modeled concentrations and the measured.

S10 Multimedia exposure model

Humans are exposed to dioxins via three main routes, inhalation, ingestion, and dermal contact with contaminated substances, leading to body burden of dioxins[50]. Exposure pathways considered in this assessment include inhalation through air and airborne particles, ingestion of cereal, vegetable, edible oil, fruit, fish, meat, milk, egg, and drinking water, dermal contact with airborne particles, soil, and water.

The average daily doses (ADDs) were used to estimate human exposure to 2,3,7,8-TCDD[51],

$$ADD = \frac{C_i \times EIR \times EF \times ED}{BW \times AT}$$

where C_i is the contaminant concentration in exposure medium *i*. *BW* is the body weight of receptors. *EIR* is the efficient intake rate of 2,3,7,8-TCDD via different exposure pathway. *EF* is exposure frequency. *ED* is exposure duration. BW is the body weights of receptors. *AT* is average lifespan.

For inhalation, *EIR* can be calculated as follows:

$$EIR = IR \times f_r$$

where *IR* is the air intake rate. f_r is retention coefficient in lung.

EIR of ingestion is defined by

$$EIR = FC \times ABS$$

where *FC* is the consumption coefficient for various food. The dietary patterns of Chinese residents are illustrated in **Fig. S1** using the data reported by NBSC[52]. *ABS* is gastrointestinal

absorption factor of PCDD/F.

For dermal contact exposure, four pathways via air, airborne particles, water and soil were taken into consideration. The *EIR* from dermal contact exposure to air is defined by

$$EIR = TE \times k \times SA$$

where *TE* is exposure duration to water. *k* is absorption coefficient of skin. *SA* is human body surface areas.

The *EIR* from dermal contact exposure to soil is defined by

$$EIR = SSA \times f \times ESA$$

where *SSA* is soil-to-skin adherence rate. *f* is intake fraction of soil. *ESA* is efficient surface area of body. The *EIR* from dermal contact exposure to airborne particles is also calculated by the above equation.

The total exposure doses (TEDs, pg/kg bw/day) were estimated by the sum of the intakes via inhalation, ingestion, and dermal exposure.

The per capita annual consumption of various food for different scenarios from 1980 to 2009 were shown as **Fig. S1**. Due to absence of data before 1990, it was assumed that consumption of fruits and milk from 1980 to 2009 was same as that in 1990. For input of model, annual food consumption were converted to daily consumption. Other parameters used in the exposure model were listed in **Table S10**.

Table S10 Exposure factors used in the model

Parameters	Default value	*CV* (%)	Ref	*Cf*
Body weight (kg)	65	17	51	1.4
Air intake rate (IR, m^3/day)	15.2	44	51	2.4
Drinking water intake (liter/day)	2.0	53	51	2.8
Lifetime (AT, years)	70	13	51	1.3
Efficient intake rate (EIR)				
for inhalation	100%		51	
for ingestion	100%		53	
for dermal contact (bath water) (cm/hr)	1.4×10^{-2}	17	54	1.4
for dermal contact (airborne particles)	1.0%	21	55	1.5
for dermal contact (soil)	1.5%	16	51	1.4
Efficient surface area of body (ESA, m^2)	0.312	14	51	1.3

Human body surface areas (A_r, m^2)	2.0	10	51	1.2
Exposure duration to water (*TE*, min/day)	10	22	51	1.6
Soil-to-skin adherence rate (*SSA*, mg/cm^2)	0.425	21	51	1.5
Retention coefficient in lung (f_r)	0.75	30	56	1.8
Gastrointestinal absorption factor (ABS)	1.0	30	57	1.8
Absorption coefficient of skin (k, cm/h)	0.001	30	58	1.8
Exposure frequency (d/yr)	365		50	
Exposure duration (ED, d)	25550		50	
Average lifespan (days)	70	28	50	1.8

References

(1) Kalnay, E. et al. The NCEP/NCAR reanalysis 40-year project. *Bull. Am. Meteorol. Soc.* **77,** 437-471 (1996).

(2) Ma, J., Daggupaty, S., Harner, T. & Li, Y. Impacts of lindane usage in the Canadian Prairies on the Great Lakes ecosystem. 1. Coupled atmospheric transport model and modeled concentrations in air and soil. *Environ. Sci. Technol.* **37,** 3774-3781 (2003).

(3) Mackay, D., Shiu, W. Y., Ma, K. C. & Lee, S. C. Handbook of physical-chemical properties and environmental fate for organic chemicals; CRC Press: Boca Raton (2006).

(4) Macleod, M., Fraser, A. J. & Mackay, D. Evaluating and expressing the propagation of uncertainty in chemical fate and bioaccumulation models. Environmental Toxicology and Chemistry, **21,** 700-709 (2002).

(5) Huang, T. et al. Gridded atmospheric emission inventory of 2,3,7,8-TCDD in China. *Atmos. Environ.* **108,** 41-48 (2015).

(6) Harrad, S. & Smith, D. J. T. Evaluation of a terrestrial food chain model for estimating foodstuff concentration of PCDD/Fs. *Chemosphere* **34,** 1723-1737 (1997).

(7) Ma, J., Venkatesh, S., Li, Y. & Daggupaty, S. M. Tracking toxaphene in the North American Great Lakes basin-1. Impact of toxaphene residues in the U.S. soils. *Environ. Sci. Technol.* **39,** 8132-8141 (2005).

(8) Zhang, L., Ma, J., Venkatesh, S., Li, Y. & Cheung, P. Modeling evidence of episodic intercontinental long-range Transport of Lindane. *Environ. Sci. Technol.* **42,** 8791-8797 (2008).

(9) Zhang, Y., Tao, S., Shen, H. & Ma, J. Inhalation exposure to ambient polycyclic aromatic hydrocarbons and lung cancer risk of Chinese population. *Proc. Natl. Acad. Sci.* **106,** 21063-21067 (2009).

(10) Zhang, L., Ma, J., Tian, C. & Li, Y. Atmospheric transport of persistent semi-volatile organic chemicals to the Arctic and cold condensation in the mid-troposphere: Part 2. 3D modeling of episodic atmospheric transport. *Atmos. Chem. Phys.* **10,** 7315-7324 (2010).

(11) Lehndorff, E. & Schwark, L. Biomonitoring airborne parent and alkylated three-ring PAHs in the Greater Cologne Conurbation I: Temporal accumulation patterns. *Environ. Pollut.* **157,** 1323-1331 (2009).

(12) Tremolada, P., Burnett, V., Calamari, D. & Jones, K, C. A study of the spatial distribution of PCBs in the UK atmosphere using pine needles. *Chemosphere* **32,** 2189-2203 (1996).

(13) Ockenden, W. A., Steinnes, E., Parker, C. & Jones, K. C. Observations on persistent organic pollutants in plants: Implications for their use as passive air samplers and for POP cycling. *Environ. Sci. Technol.* **32,** 2721-2726 (1998).
(14) Romanic, S. H. & Krauthacker, B. Distribution of persistent organochlorine compounds in one-year and two-year-old pine needles. *Bull. Environ. Contam. Toxicol.* 77, 143-148 (2006).
(15) Rappolder. M., Schroter, C., Schadel, S., Waller, U. & Korner, W. Temporal trends and spatial distribution of PCDD, PCDF, and PCB in pine and spruce shoots. *Chemosphere* **67,** 1887-1896 (2007).
(16) Chen, P., Mei, J., Peng, P., Hu, J. & Chen, D. Atmospheric PCDD/Fs Concentrations in 38 Cities of China Monitored with Pine Needles, a Passive Biosampler. *Environ. Sci. Technol.* **46,** 13334-13343 (2012).
(17) Sun, J. Atmospheric pollution profiles, gas-particle partitioning of polychlorinated dibenzo-p-dioxins and dibenzo-furans (PCDD/Fs) and polychlorinated biphenyls (PCBs) in Beijing. Ph. D. Dissertation, Beijing: China University of Geosciences (2009).
(18) Ding, L. et al. Seasonal trend of ambient PCDD/Fs in Tianjin City, northern China using active sampling strategy. *J. Environ. Sci. China* **24,** 1966-1971 (2012).
(19) Chen, T., Li, X., Yan, J., Lu, S. & Cen, K. Distribution of polychlorinated dibenzo-p-dioxins and dibenzofurans in ambient air of different regions in China. *Atmos. Environ.* **45,** 6567-6675 (2011).
(20) Li, Y., Jiang, G., Wang, Y., Cai, Z. & Zhang, Q. Concentrations, profiles and gas-particle partitioning of polychlorinated dibenzo-p-dioxins and dibenzofurans in the ambient air of Beijing, China. *Atmos. Environ.* **42,** 2037-2047 (2008).
(21) Ying, Y. Preliminary study on levels of PCDD/Fs in typical areas atmosphere and soils of Shanghai, MS Dissertation. MS Dissertation, East China University of Science and Technology, Shanghai (2010).
(22) Yu, L. Preliminary study on levels of PCDD/Fs in atmosphere of Guangzhou and Typical emission sources of PCDD/Fs, China. Doctoral dissertation, Guangzhou Institute of Geochemistry, Chinese Academy of Science, Guangzhou (2007).
(23) Wang, W. et al. Pollution level and distribution of PCDD/PCDF congeners between vapor phase and particulate phase in winter air of Dalian, China. *J. Environ. Sci.* **23,** 36-39 (2011).
(24) Ren, Z. et al. Characteristics of air pollution by polychlorinated dibenzo-p-dioxins and dibenzofurans in the typical industrial areas of Tangshan City, China. *J. Environ. Sci.* **23,** 228-235 (2011).
(25) Wang, C. Study on contamination levels on dibenzo-p-dioxins and dibenzofurans, polychlorinated biphentls and polybrominated diphenyl ethers in atmosphere of a seaside city, China. MS Dissertation, Southwest University, Chongqing (2010).
(26) Yang, Z., Ni, Y., Zhang, Q., Chen, J. & Liang, X. Research on PCDD/Fs contents in several atmospheric aerosol and soil samples collected at Shanghai and Dalian. *Guangzhou Environ. Sci.* **19,** 25-27 (2004).
(27) Yang, Y. Altitude gradient distributions of dioxin-like compounds and PCNs in soils from Wolong area and human health risk assessment for yak consumption. *Environ. Chem.* **28,** 276-283 (2009).
(28) Luo, Y. et al. Soil environmental quality and remediation in Yangtze river delta region 1. Composition and pollution of polychlorinated dibenzo-p-dioxins and dibenzofurans

(PCDD/Fs) in a typical farmland. *Acta Pedologica Sinica* **42,** 570-576 (2005).
(29) Han, J., Shen, H., Yu, C., Song, G. & Ma, B. Primary investigation of PCDD/Fs and PCBs in soils from some areas in Zhejiang. Chin. *J. Health Lab. Technol.* **19,** 1124-1127, (2009).
(30) Anna, O. W., William, J., Anthony, S. & Ming, H. Spatial distribution of polybrominated diphenyl ethers and polychlorinated dibenzo-p-dioxins and dibenzofurans in soil and combusted residue at Guiyu, an electronic waste recycling site in Southeast China. *Environ. Sci. Technol.* **41,** 2730-2737 (2007).
(31) Liu, X. et al. The distribution characteristics of PCDD/Fs in surface soils from different areas of Baiyin city, China. *J. Agro-Environ. Sci.* **29,** 1137-1143 (2010).
(32) Zemba, S. G., Green, L. C., Crouch, E. A. C. & Lester, R. R. Quantitative risk assessment of stack emissions from municipal waste combustors. *J. Hazard. Mater.* **47,** 229-275 (1996).
(33) Lorber, M. et al. Development and validation of an air-to-beef food chain model for dioxin-like compounds. *Sci. Tot. Environ.* **136,** 39-65 (1994).
(34) NYSDOH (New York State Department of Health). Guidance for Exposure Assessment of Municipal Solid Waste and Hospital Waste and Hospital Waste Incinerator Emissions. New York State Department of Health, Albany, NY (1991).
(35) Wagrowski, D. M. & Hites, R. A. The Accumulation of Polychlorinated Dibenzo-p-dioxins and Dibenzofurans in the Food Chain. *Organohalogen Compounds* **32,** 233-237 (1997).
(36) Lim, Y., Yang, J., Kim, Y., Chang, Y. & Shin, D. Assessment of human health risk of dioxin in Korea. *Environ. Monit. Assess.* **92,** 211-228 (2004).
(37) La, R. C. et al. TEQs and body burden for PCDDs, PCDFs, and dioxin-like PCBs in human adipose tissue. *Chemosphere* **73,** 92-96 (2008).
(38) Yang, Y., Wang, G. & Pan, X. China food composition; Peking University Medical Press, Beijing (2009).
(39) Xu, Y. et al. Assessing cancer risk in China from gamma- hexachlorocyclohexane emitted from Chinese and Indian sources. *Environ. Sci. Technol.* **37,** 3774-3781 (2013).
(40) Jiang, J. et al. Determination of PCDDs/ PCDFs in fish by isotope dilution HRGC/ HRMS. *Chin. Prev. Med.* **5,** 437-440 (2004).
(41) Zhang, J. et al. Level of polychlorinated dibenzo-p-dioxins and dibenzo-furans in sea fish samples in some sea areas in China. *Chin. Prev. Med.* **39,** 253-256 (2005).
(42) Jiang, Y. et al. Analyzed of PCDD /Fs, PCBs and PBDEs in fish sample. *Chin. J. Health Lab. Technol.* **20,** 1631-1635 (2010).
(43) Han, J., Shen, H. & Tie, X. Level of polychlorinated dibenzo-p-dioxin and dibenzofuran concentrations in freshwater fish in Northwestern, Zhejiang. *Chin. J. Health Lab. Technol.* **16,** 546-548 (2006).
(44) Zhang J. et al. Polychlorinated dibenzo-p-dioxin and dibenzofuran concentrations in common fish species in the Pearl River Delta area, China. *Chemosphere* **66,** 199-202 (2007).
(45) Gao, L., Zhang, Q., Zhang, B., Liu, W. & Xiao, K. Polychlorinated dibenzo-p-dioxins and dibenzofurans in water and six fish species from Dongting Lake, China. *Chemosphere* **114,** 150-157 (2014).
(46) Han, J. Dioxin and PCBs in Zhejiang: Pollution status and their risk assessment to human health. Doctoral dissertation, Zhejiang University, Hangzhou (2011).
(47) Shen, H. et al. Levels and congener profiles of PCDD/Fs, PCBs and PBDEs in seafood from China. *Chemosphere* **77,** 1206-1211 (2009).

(48) Zhang, Q. & Jiang, G. Polychlorinated dibenzo-p-dioxins/furans and polychlorinated biphenyls in sediments and aquatic organisms from the Taihu Lake, China. *Chemosphere* **61,** 314-322 (2005).

(49) Liu, B. et al. Contamination of polychlorinated dibenzo-p-dioxins and polychlorinated dibenzo-p-furans in poultry products in Shenzhen. *Chin. J. Food Hyg.* **22,** 6-10 (2010).

(50) Esteban, M. & Castaño, A. Non-invasive matrices in human biomonitoring: a review. *Environ. Int.* **35,** 438-49 (2009).

(51) U.S. EPA (the United States Environmental Protection Agency). Exposure Factors Handbook; Environmental Protection Agency: Washington D. C. (1997).

(52) NBSC (National Bureau of Statistics of China). China statistical yearbook 2010. China Statistics Press, Beijing (2010).

(53) Berg van den B. Human exposure to soil contamination: a qualitative and quantitative analysis towards proposals for human toxicological intervention values (partly revised edition); National Institute of Public Health and Environmental Protection: Netherlands (1994).

(54) U.S. EPA (the United States Environmental Protection Agency). Dermal exposure assessment: principles and applications; Environmental Protection Agency: Washington D. C. (1992).

(55) Guo, M. et al. Population exposure to HCH in Tianjin area. *Environ. Sci.* **2,** 164-167 (2005).

(56) Hu, X. B., Xu, Z C., Wang, J. N. & Zhang, X. Y. Human health potential impacts of polychlorinated dibenzo-p-dioxins and dibenzofurans pollution in Pearl River Delta. *Ecol. Environ. Sci.* **20,** 311-316 (2011).

(57) Karademir, A. Health risk assessment of PCDD/F emissions from a hazardous and medical waste incinerator in Turkey. *Environ. Int.* **30,** 1027-1038 (2004).

(58) U.S. EPA (the United States Environmental Protection Agency). Exposure and Human Health Reassessment of 2, 3, 7, 8-TCDD and Related Compounds Part I, Vol. 3. Properties, Environmental Levels, and Background Exposures, Environmental Protection Agency, Washington D. C. (2000).
